# Ferroptosis‐Mediated Hippocampal Neuronal Loss Post‐mTBI: Chromatin Accessibility Profiling and Single‐Nucleus Transcriptomics

**DOI:** 10.1002/advs.202512362

**Published:** 2025-12-15

**Authors:** Manrui Li, Qiuyun Yang, Shengqiu Qu, Yang Chen, Yang Shen, Yang Xu, Xilong Lin, Yihan Sun, Ying Chen, Meili Lv, Lin Zhang, Zengqiang Yuan, Weibo Liang, Xiameng Chen

**Affiliations:** ^1^ Department of Forensic Genetics West China School of Basic Medical Sciences and Forensic Medicine Sichuan University Chengdu 610041 China; ^2^ Department of Forensic Pathology and Forensic Clinical Medicine West China School of Basic Medical Sciences and Forensic Medicine Sichuan University Chengdu 610041 China; ^3^ Department of Immunology West China School of Basic Medical Sciences and Forensic Medicine Sichuan University Chengdu 610041 China; ^4^ West China Second Hospital Sichuan University Chengdu Sichuan 610041 China; ^5^ Beijing Institute of Basic Medical Sciences Beijing 100850 China

**Keywords:** ferroptosis, mild TBI, snRNA‐seq, Tmsb4x

## Abstract

Neuronal death in the hippocampus following mild traumatic brain injury (mTBI) is a hallmark of cognitive dysfunction, yet the underlying molecular mechanisms remain poorly understood. To address this issue, the hippocampus of eCCI‐modeled mTBI mice is profiled using single‐nucleus RNA‐seq and ATAC‐seq, producing matched, cell‐type‐resolved transcriptomic and chromatin‐accessibility datasets. Gene sets covering major cell‐death pathways are curated and scored for their activity across neuronal subtypes. Ferroptosis emerged as the dominant program. Gene‐set enrichment indicated activation of mitochondrial damage‐related pathways in neurons, a hallmark of ferroptosis. Transcription‐factor analyses, supported by epigenetic data, showed decreased binding of c‐Jun and Rfx3 in dentate granule cells. Functional assays show that the c‐Jun‐regulated gene Tmsb4x protects hippocampal neurons after mTBI. It downregulates Slc2a2, counteracts ferroptosis, and is associated with improved motor and cognitive performance in mice. This study advances understanding of mTBI pathogenesis at single‐cell resolution, identifying ferroptosis as a critical determinant of neuronal death and uncovering *Tmsb4x* as a promising therapeutic target for mitigating cognitive dysfunction.

## Graphical abstract

Hippocampal single ‐nucleus transcriptomes and chromatin accessibility after mild traumatic brain injury reveal dentate granule neuron vulnerability driven by ferroptosis. The c‐Jun–Tmsb4x–Slc2a2 axis modulates lipid peroxidation and iron dysregulation. Functional assays—including ChIP‐qPCR, in vitro ferroptosis rescue, and TEM—corroborate mechanisms and highlight Tmsb4x/Tβ4 as potential neuroprotective avenues.

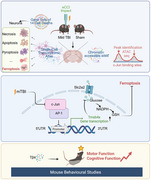

## 1 Introduction

Mild traumatic brain injury (mTBI) refers to the acute disruption of brain function caused by a blow or impact to the head, accounting for ≈80% of all traumatic brain injuries.^[^ ^1^ ^]^ It is a common neurological injury encountered in clinical practice. mTBI can induce pathophysiological changes in the brain, leading to acute neurological deficits and a range of subsequent acute or chronic sequelae.^[^ ^2^ ^]^ Patients with mTBI often experience a variety of physical and cognitive symptoms, including headaches, dizziness, nausea, memory deficits, difficulty concentrating, reduced learning ability, sleep disturbances, depression, and anxiety.^[^ ^3^ ^]^ Cognitive dysfunction is a prevalent sequela among mTBI patients, lasting from several months to years, and can even result in permanent impairment, significantly impacting the patients' daily social functioning.^[^ ^4^ ^]^

mTBI presents a wide range of clinical symptoms that reflect the complex pathophysiological changes occurring in brain cells post‐injury. Different brain regions and cell types play distinct roles during the injury and repair processes.^[^ ^5^ ^]^ Notably, hippocampal dysfunction is closely associated with cognitive impairments following mTBI.^[^ ^6^ ^]^ The hippocampus supports learning and memory through circuits that interact with multiple brain regions.^[^ ^7^ ^]^ Damage or loss of hippocampal neurons is common after mTBI and contributes to cognitive deficits.^[^ ^8^ ^]^ Understanding the molecular basis of changes in the hippocampus after mTBI is essential for elucidating its pathogenesis and identifying effective intervention targets.

Past studies have primarily focused on the overall damage and death of hippocampal neurons.^[^ ^9^, ^10^ ^]^ However, due to the high heterogeneity of neurons, such broad analyses may overlook the specific responses of different neuronal subtypes following injury. For instance, Vadim Tashlykov et al. observed diffuse neuronal damage and apoptosis after mTBI, with the most severe injuries occurring in the anterior cingulate cortex and the hippocampal CA3 region.^[^ ^9^, ^10^ ^]^ In contrast, Tian et al. did not find significant apoptosis of hippocampal neurons in mTBI mice, suggesting that hippocampal‐related cognitive dysfunction is mainly due to neurodegeneration associated with upregulation of neuronal p‐tau.^[^ ^11^ ^]^ The differing conclusions from these overall analyses of hippocampal damage may affect our understanding of the specific pathophysiological mechanisms occurring in the hippocampus post‐mTBI. Refined approaches, such as single‐nucleus RNA‐seq (snRNA‐seq), can resolve subtype‐specific transcriptional changes after mTBI. snRNA‐seq provides the cell‐type detail needed to interpret hippocampal injury.

This study employs snRNA‐seq to thoroughly analyze the transcriptional changes in hippocampal neurons at the single‐cell level following mTBI in mice. Specifically, it investigates the activation of cell death pathways and key regulatory molecules in different neuronal subtypes after injury. By exploring the roles and mechanisms of protective molecules in neuronal damage, this research aims to identify new targets and strategies for treating cognitive dysfunction in mTBI patients.

## 2 Results

### 2.1 Single‐Cell Transcriptomics Reveals Ferroptosis as a Key Cell Death Pathway in Hippocampal Neurons Following mTBI

**Figure 1:**
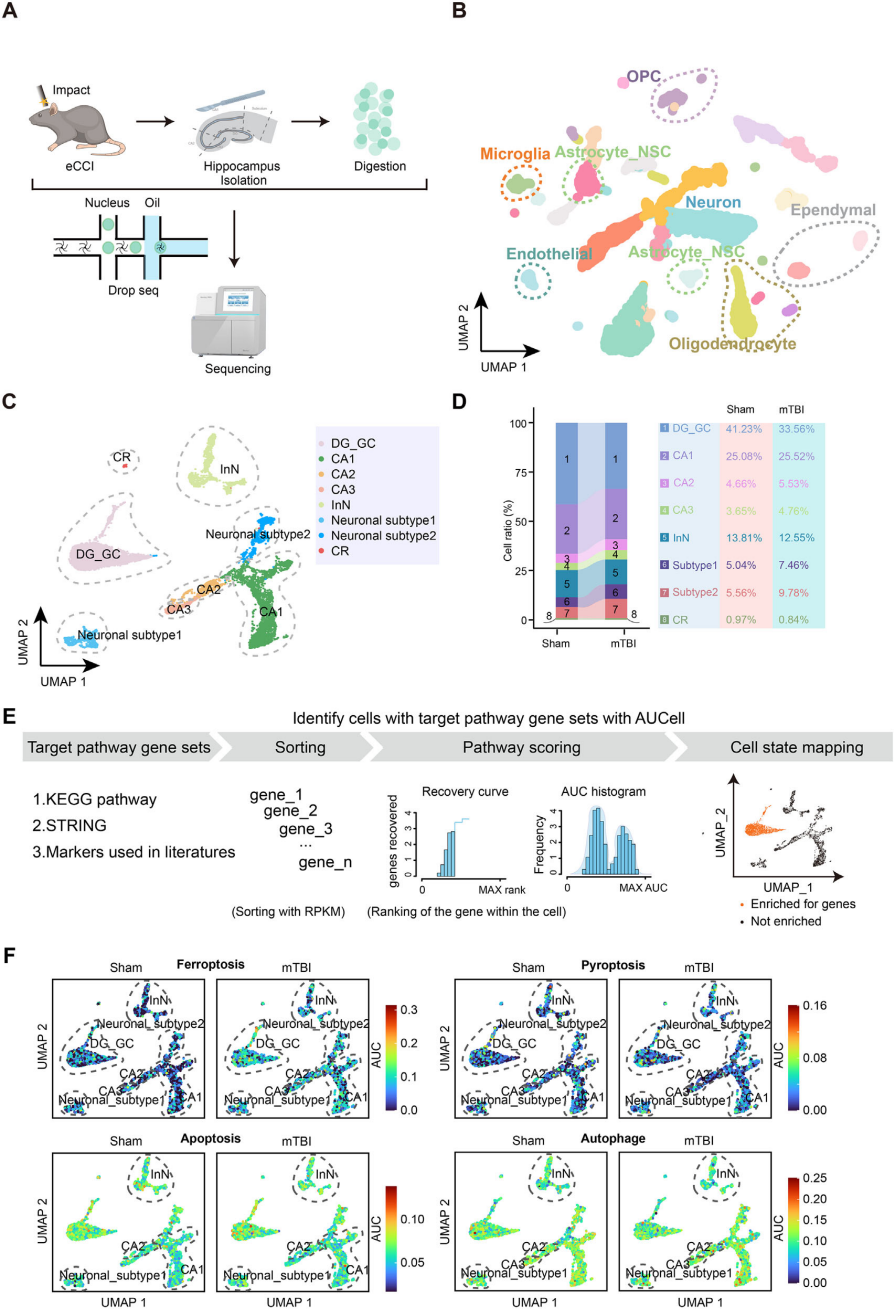
Single‐nucleus RNA sequencing of the hippocampus in mTBI mice revealed the activation of the ferroptosis pathway in neurons. A) Schematic diagram illustrating hippocampus sampling, single‐nucleus sequencing, and analysis following mTBI. B) UMAP clustering of seven cell populations (12,478 nuclei). C) UMAP clustering of eight neuronal subtypes. D) Stacked bar chart showing the proportions of neuronal subtypes in the mTBI and sham groups (Table S4, Supporting Information). E) Schematic representation of the compilation of cell death pathway gene sets and activation scoring based on AUC analysis. F) UMAP visualization of activation scores for ferroptosis, pyroptosis, apoptosis, and autophagy pathways, with dashed lines indicating neuronal subtypes showing increased activation post‐mTBI. Abbreviations: mTBI, mild traumatic brain injury; UMAP, uniform manifold approximation and projection; AUC, area under the curve.

We obtained high‐quality single‐nucleus transcriptomic data from 12,478 nuclei of hippocampal samples from the mTBI and sham groups using snRNA‐seq (**Figures** **1**A; S1A–F, Supporting Information). We characterized the cell identities of the clusters based on cell‐specific markers: 1) Neurons expressing *Meg3, Gria1, and Syt1*; 2) Oligodendrocytes expressing *Mbp, Plp1*, and *Mobp*; 3) Astrocyte_NSCs expressing *Slc1a3, Mertk*, and *Aqp4*; 4) Oligodendrocyte precursor cells (OPCs) expressing *Pdgfra*; 5) Microglia expressing *Csf1r* and *Cx3cr1*; 6) Ependymal cells expressing *Dnah11*; and 7) Endothelial cells expressing *Flt1* and *Rgs5* (Figures 1B; S2A–C and Tables S1 and S2, Supporting Information).

To investigate the specific transcriptional changes in hippocampal neurons following mTBI, we isolated and further subdivided hippocampal neurons. Using known hippocampal neuron subtypes and their specific markers, we classified them into eight subtypes: *Prox1* ^+^ dentate gyrus granule cells (DG_GC), *Mpped1* ^+^ CA1 neurons, *Necab2* ^+^ CA2 neurons, *Nectin3* ^+^ CA3 neurons, *Gad2* ^+^ inhibitory neurons (InN), *Reln* ^+^ Cajal‐Retzius cells (CR), Neuronal subtype 1, and Neuronal subtype 2 (Figures 1C; S3A–C and Table S3, Supporting Information). We found that the number of DG_GC cells significantly decreased after mTBI and that pathways related to axonogenesis were activated (Figures 1D; S2D and Table S4, Supporting Information). To further explore the mechanisms underlying DG_GC loss, we integrated markers for all known modes of cell death and constructed a gene set encompassing 14 distinct cell death pathways, including traditional forms and newer forms (Figure 1E and Table S5, Supporting Information).^[^ ^11^, ^12^, ^13^, ^14^ ^]^ We then calculated the activation scores for cell death pathways in different neuronal subtypes after mTBI through gene ranking and area under the curve (AUC) analysis, providing the transcriptional features of cell death in hippocampal neurons (Figure 1E). The results revealed that different neuronal subtypes activate distinct cell death pathways. Even within the same neuronal subtype, multiple cell death pathways can be triggered, reflecting the complexity of neuronal damage patterns after injury (Figures 1F; S3D and Table S6, Supporting Information). Notably, ferroptosis was activated in nearly all neuronal subtypes, with activation scores significantly higher than those of other cell death pathways (Figure 1F and Table S6, Supporting Information). Moreover, DG_GC showed a significant increase in ferroptosis activity in mTBI versus sham with the largest effect size among subtypes (Figure S3E, Supporting Information). Consistent with prior work linking ferroptosis to TBI‐induced hippocampal injury, including the DG, our single‐cell analysis delineates this vulnerability at subtype resolution.^[^ ^15^ ^]^ These findings suggest that ferroptosis activation may be a critical feature of hippocampal fate regulation following mTBI.

### 2.2 Changes in Chromatin Accessibility of Mitochondrial Damage‐Related Genes Induced by c‐Jun and Rfx3 Transcriptional Regulation are Key Mechanisms of Neuronal Ferroptosis in mTBI

**Figure 2:**
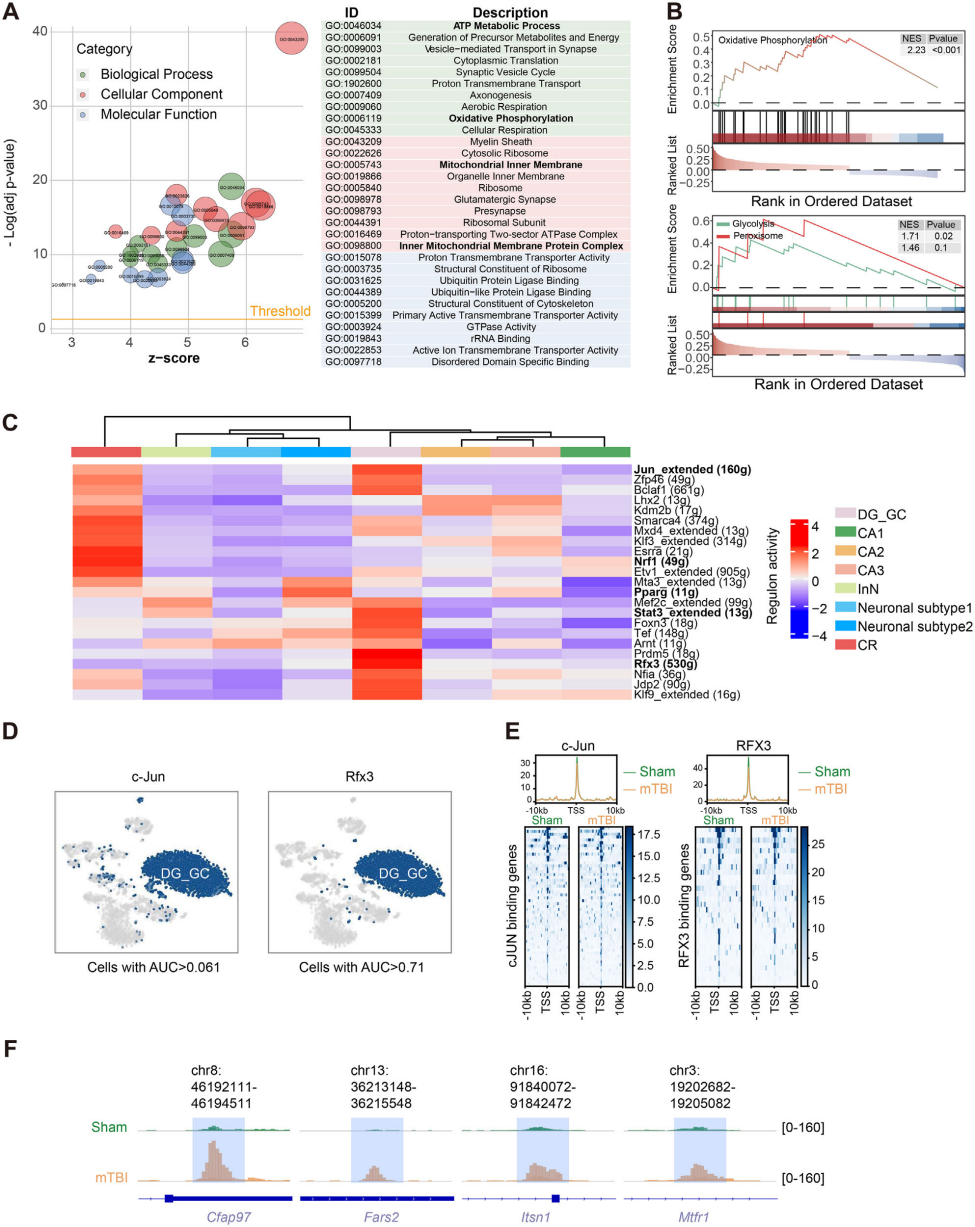
Analyses reveal mitochondrial damage pathways and transcription factor activation in neurons. A) Bubble plot showing enriched GO terms for DEGs in neurons, categorized by Biological Process, Cellular Component, and Molecular Function. Pathways related to mitochondrial damage are highlighted in bold. B) GSEA plot illustrating the enrichment of oxidative phosphorylation‐related genes in hippocampal neurons of mTBI mice. C) Expression of the mitochondrial damage‐related gene Kit in single cells, with expression localized primarily in neurons. D) SCENIC heatmap displaying changes in the activity of transcription factors in neurons. E) UMAP plots from SCENIC‐based reclustering of neurons by regulon activity, showing activation of transcription factors c‐Jun and Rfx3 in DG_GC cells. F) Chromatin accessibility of genes regulated by transcription factors c‐Jun and Rfx3, as detected by ATAC sequencing. The analysis focuses on regions within ±10 kb of the TSS. Abbreviations: GO: Gene Ontology; DEGs: Differentially Expressed Genes; mTBI: mild Traumatic Brain Injury; SCENIC: Single‐Cell Regulatory Network Inference and Clustering; DG_GC: Dentate Gyrus Granule Cell; ATAC‐seq: Assay for Transposase‐Accessible Chromatin using sequencing; TSS: Transcription Start Site.

We further investigated the mechanisms underlying energy metabolism abnormalities and mitochondrial damage related to ferroptosis. Pathways related to energy metabolism and mitochondrial damage, including oxidative phosphorylation, glycolysis, and lipid metabolism, were activated (**Figures** **2**A,B; S4A, Supporting Information). These pathways are closely linked to mitochondrial function, the impairment of which is a central feature of ferroptosis.^[^ ^16^ ^]^ Consequently, our results further support the involvement of ferroptosis in neuronal loss following mTBI.

Building on these findings, we employed the SCENIC approach to explore the transcriptional regulatory mechanisms underlying the mitochondrial damage in neurons. We observed significant alterations in the activity of transcription factors associated with mitochondrial damage (Figure 2C). Notably, the regulatory activity of c‐Jun and Rfx3 exhibited specific changes in the DG_GC neurons, which experienced the greatest loss (Figures 2D; S4B, Supporting Information), indicating that these transcription factors may play critical roles in the loss of DG_GC after injury. Interestingly, the decreased regulatory activity of Rfx3 was primarily due to reduced mRNA levels, while no similar phenomenon was observed in c‐Jun transcripts. This leads us to hypothesize that c‐Jun mainly regulates downstream gene expression by modulating the binding strength to target promoters (Figure S4C, Supporting Information). To test this hypothesis, we performed ATAC sequencing to analyze the chromatin accessibility of mitochondrial damage‐related genes, thereby examining epigenetic changes in transcriptional regulation following mTBI. The results indicated that the binding strength of c‐Jun and Rfx3 significantly decreased in the mTBI group (Figure 2E), consistent with the single‐cell sequencing findings. Furthermore, we observed increased accessibility of downstream genes related to mitochondrial damage and function (e.g., Cfap97, Fars2, Itsn1, Mtfr1) (Figure 2F; Table S7, Supporting Information).^[^ ^17^, ^18^, ^19^, ^20^ ^]^ This increase aligns with the negative transcriptional regulation exerted by c‐Jun and Rfx3 on multiple downstream genes, corroborating previous research findings.^[^ ^21^, ^22^ ^]^ Analysis of the ATAC sequencing and snRNA‐seq data collectively highlights the critical role of c‐Jun and Rfx3 in the transcriptional regulation of mitochondrial damage following mTBI.

### 2.3 c‐Jun‐Mediated Upregulation of Tmsb4x Expression in Neurons is a Key Feature of Neuronal Damage in mTBI

**Figure 3:**
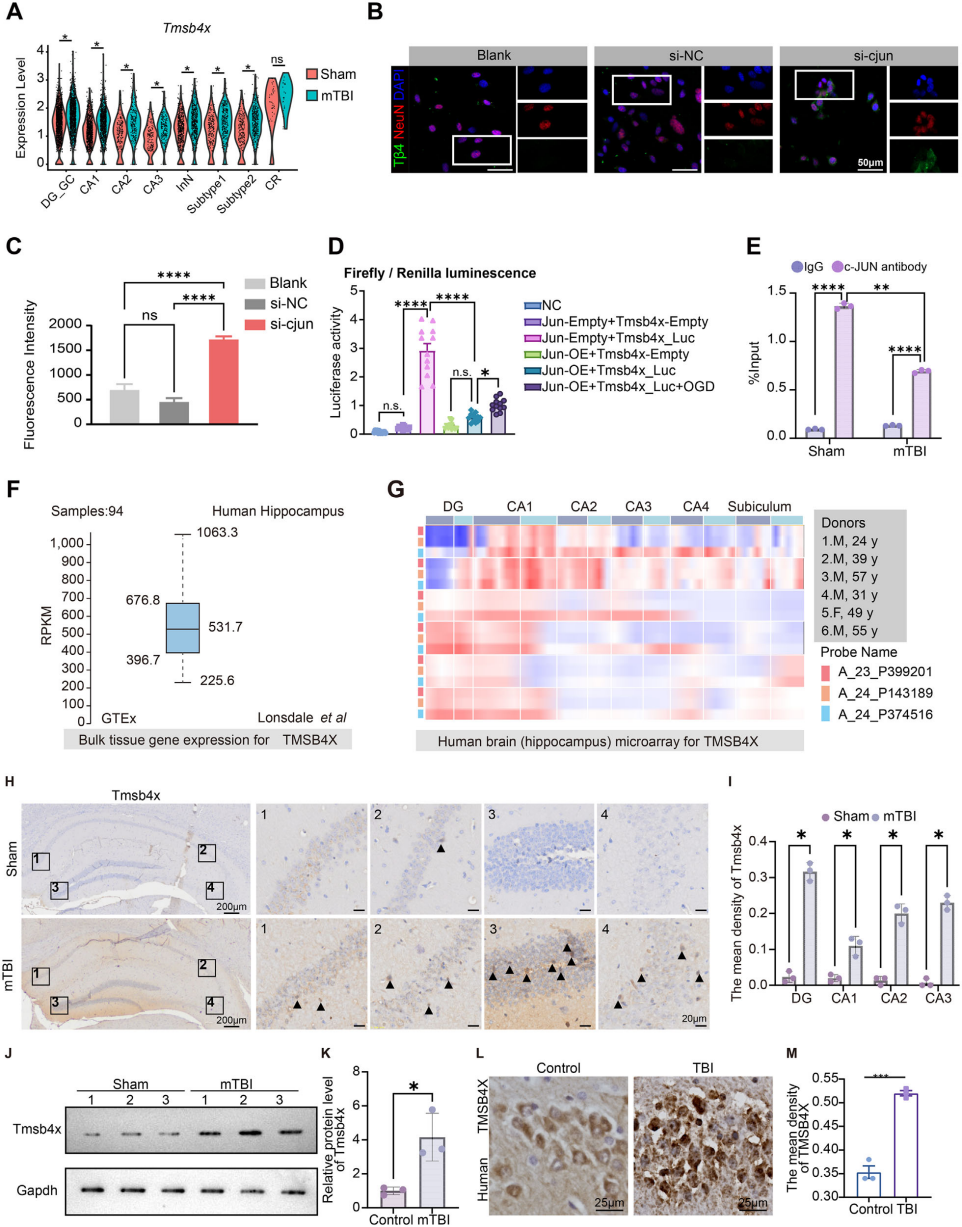
Activation and elevated expression of Tmsb4x in neurons post‐brain injury mediated by transcription factor c‐Jun. A) snRNA‐seq violin plot showing Tmsb4x expression across neuronal subtypes in the mTBI and sham groups. The levels of significance were set at *p* < 0.05 (*), n.s. = non‐significant. B) Immunofluorescence of c‐Jun in neurons under the indicated conditions (Blank, si‐NC, si‐c‐jun); nuclei, DAPI. C) Bar plot quantifying the intensity of the Tβ4 protein signal (488 nm) from immunofluorescence staining in the in vitro experiment (*n* = 3/group). Data are represented as the mean ± SEM. *p*‐values are calculated using one‐way ANOVA with Bonferroni correction, ****p‐value < 0.0001. D) Tmsb4x promoter luciferase assay under c‐Jun overexpression/knockdown and OGD as indicated; Firefly/Renilla ratio (*n* = 12/group). The data are represented as the mean ± SEM. One‐way ANOVA with Tukey's post hoc test was used, *p‐value < 0.05, ****p‐value < 0.0001. E) ChIP‐qPCR for c‐Jun at the Tmsb4x promoter in hippocampus (sham vs mTBI, 48 h). Signals were normalized to percent input and expressed relative to IgG (*n* = 3/group). The data are presented as means ± SEM. Two‐way ANOVA with Sidak's multiple comparisons test was used, **p‐value < 0.01, ****p‐value < 0.0001. F) Expression levels of TMSB4X in the human hippocampus from the GTEx database. G) Expression levels of TMSB4X across different hippocampal regions in the human brain as detected in a microarray database. H) Mouse hippocampal Tmsb4x IHC with subregion marked (boxes 1–4). I) Subregion‐resolved quantification of Tmsb4x‐positive cell density within the indicated hippocampal subregions (per‐animal points shown, *n* = 3/group). Shown are mean values ± SEM. *p*‐values measured by two‐way ANOVA with Sidak's multiple comparisons test, *p‐value < 0.05. J) Western blot of Tmsb4x in mouse hippocampus; Gapdh as loading control. K) Densitometric quantification of Tmsb4x/Gapdh (*n* = 3/group). Shown are mean values ± SEM from two independent experiments. *p*‐values measured by *t*‐test, *p‐value < 0.05. L,M) IHC analysis showing TMSB4X expression in human hippocampal neurons and corresponding quantification of signal intensity (*n* = 3/group). Shown are mean values ± SEM from two independent experiments. *p*‐values measured by *t*‐test, ***p‐value < 0.001. Abbreviations: mTBI, mild traumatic brain injury; IHC, immunohistochemistry.

Tmsb4x is upregulated in almost all neuronal subtypes and contains c‐Jun binding sites (https://www.genecards.org/) (**Figure** **3**A). In vitro experiments showed that Tmsb4x protein (Tβ4) levels were significantly elevated in c‐Jun knockdown neurons, indicating that c‐Jun negatively regulates Tmsb4x expression (Figure 3B,C). Luciferase assay demonstrated that c‐Jun overexpression significantly reduced Tmsb4x promoter activity, indicating that c‐Jun negatively regulates Tmsb4x transcription. Interestingly, OGD treatment led to an upregulation of Tmsb4x promoter activity (Figure 3D; Data File S1 and S2, Supporting Information), suggesting that OGD mitigated the negative regulation of Tmsb4x by c‐Jun, thereby restoring promoter activation. These findings imply that the enhanced transcription of Tmsb4x following mTBI is linked to a reduction in c‐Jun's negative regulatory effect on the Tmsb4x promoter. Chromatin immunoprecipitation followed by qPCR demonstrated c‐Jun occupancy at the Tmsb4x promoter in vivo, with lower binding at 48 h after mTBI (Figure 3E). Database searches revealed that TMSB4X is highly expressed in human hippocampal tissue, with varying expression levels across different hippocampal regions (Figure 3F,G). These findings highlight the importance of studying Tmsb4x in relation to hippocampal diseases, as it may provide new therapeutic insights.

In the mouse hippocampus, Tmsb4x immunohistochemistry revealed stronger labeling after mTBI with representative fields taken from anatomically defined subregions (Figure 3H); quantification of Tmsb4x‐positive cell density across hippocampal subregions confirmed an overall increase (Figure 3I). Consistently, western blotting of hippocampal lysates showed higher Tmsb4x protein levels post‐mTBI (Figure 3J,K). Additionally, ISH analysis confirmed that TMSB4X expression levels in the human hippocampus post‐brain injury were similar to those observed in mice, showing an upward trend (Figures 3G–J; S5, Supporting Information). This suggests that investigating the upregulation of TMSB4X in hippocampal neuronal damage and its impact on cognitive dysfunction has significant clinical implications and could offer new perspectives for the treatment of hippocampal‐related disorders.

### 2.4 Tmsb4x Knockdown Increases Sensitivity of HT22 Cells to Ferroptosis

Research has shown that the expression product of Tmsb4x, Tβ4, acts as an endogenous iron chelator with four iron (Fe^2^⁺ and Fe^3^⁺) binding domains.^[^ ^23^ ^]^ In vitro, Tβ4 enhances macrophage antioxidant capacity and inhibits glutamate‐induced ferroptosis,^[^ ^23^, ^24^ ^]^ with protection comparable to the ferroptosis inhibitor Fer‐1.^[^ ^25^ ^]^ However, the relationship between Tmsb4x and neuronal ferroptosis following mTBI has not yet been reported.

**Figure 4:**
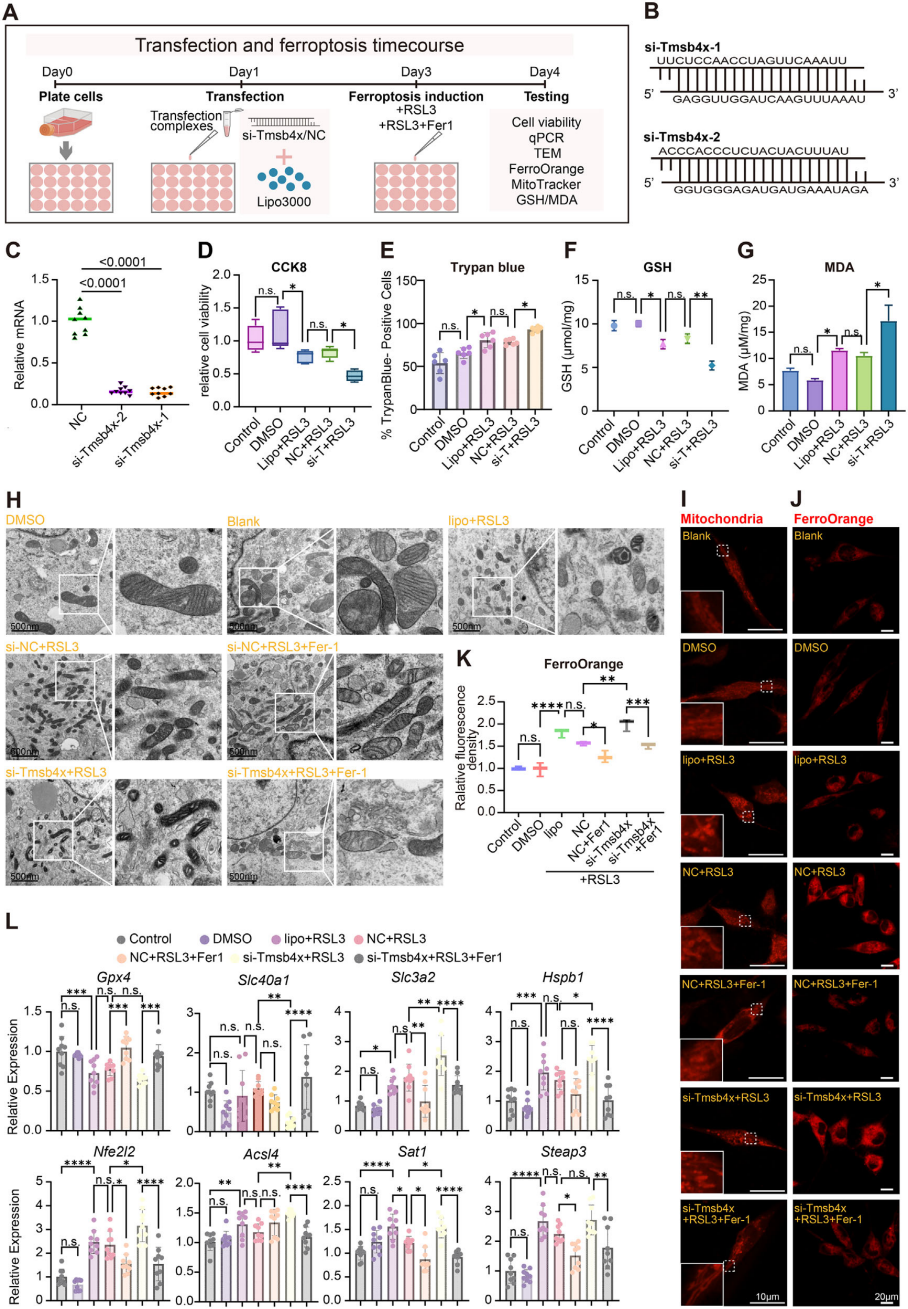
Validation of ferroptosis phenotype in Tmsb4x‐knockdown HT22 cells. Schematic diagram of the transfection process, RSL3 induction, and the timeline for ferroptosis phenotype assays in HT22 cells. B) siRNA sequences used to knock down Tmsb4x expression. C) Relative mRNA expression of Tmsb4x following siRNA knockdown in HT22 cells (*n* = 3/group). Data are represented as the mean ± SEM. *p*‐values are calculated using one‐way ANOVA with Bonferroni correction, ****p‐value < 0.0001. D) CCK‐8 viability and E) Trypan blue (% dead cells) under the indicated conditions (*n* = 5/group). The data are represented as the mean ± SEM. One‐way ANOVA with Tukey's post hoc test was used, *p‐value < 0.05. F) GSH levels in HT22 cells, representing antioxidant capacity (*n* = 6/group). Shown are mean values ± SEM. *p*‐values measured by one‐way ANOVA with Tukey's post hoc test, *p‐value < 0.05, **p‐value < 0.01. G) MDA levels in HT22 cells, indicating lipid peroxidation levels (*n* = 9/group). Shown are mean values ± SEM. *p*‐values measured by one‐way ANOVA with Tukey's post hoc test, *p‐value < 0.05. H) Transmission electron micrographs of HT22 cells processed with TEM. For each indicated condition, representative fields are shown with boxed areas displaying higher‐magnification views of mitochondria. Scale bars, as indicated. I) Mitotracker staining showing mitochondrial morphology in HT22 cells under different treatment conditions, with DAPI staining marking nuclei and mitochondrial markers highlighting mitochondrial structures (scale bar = 10 µm). J) FerroOrange staining showing iron ion accumulation in neurons under different treatment conditions. K) Quantification of FerroOrange fluorescence intensity indicating iron ion accumulation (*n* = 3/group). Shown are mean values ± SEM. *p*‐values measured by one‐way ANOVA with Tukey's post hoc test, *p‐value < 0.05, **p‐value < 0.01, ***p‐value < 0.001, ****p‐value < 0.0001. L) qPCR analysis of ferroptosis‐related gene expression, normalized to Gapdh, with data processed using the 2^−ΔΔCT^ method (*n* = 3/group). Data are represented as the mean ± SEM. *p*‐values are calculated using one‐way ANOVA with Bonferroni correction, *p‐value < 0.05, **p‐value < 0.01, ***p‐value < 0.001, ****p‐value < 0.0001. Abbreviations:RSL3, RAS‐selective lethal small molecule 3 (a ferroptosis inducer); CCK8, Cell Counting Kit‐8; NC, Negative Control; qPCR, Quantitative Polymerase Chain Reaction; GSH, Glutathione; MDA, Malondialdehyde.

Our previous data suggested that Tmsb4x may play a role in regulating neuronal ferroptosis after mTBI. To test whether Tmsb4x restrains ferroptosis, we first combined siRNA knockdown with RSL3 and read out viability and biochemical indices (**Figure** **4**A,B). Knockdown efficiency is shown in Figure 4C. RSL3 reduced cell viability (Figure 4D,E) accompanied by a decrease in GSH (Figure 4F) and an increase in MDA (Figure 4G); si‐Tmsb4x+RSL3 further aggravated these changes compared with NC+RSL3. We next introduced the ferroptosis inhibitor Ferrostatin‐1 (Fer‐1) to test rescue and expanded the readouts accordingly. Transmission electron microscopy (TEM) revealed condensed mitochondria with loss of cristae after RSL3, which were exacerbated by si‐Tmsb4x and alleviated by Fer‐1 (Figure 4H). Consistently, MitoTracker live‐cell imaging showed collapse of the tubular mitochondrial network with increased fragmentation under RSL3, worsened by si‐Tmsb4x and partially restored by Fer‐1 (Figure 4I). FerroOrange imaging demonstrated an increased labile Fe(II) pool with RSL3 that was further elevated by si‐Tmsb4x and partly normalized by Fer‐1 (Figure 4J,K). Finally, qPCR of ferroptosis‐related genes showed expression patterns consistent with enhanced ferroptotic stress under si‐Tmsb4x+RSL3 and rescue by Fer‐1 (Figure 4L). Together, these results indicate that reducing Tmsb4x heightens ferroptotic vulnerability, while pharmacologic inhibition of ferroptosis restores biochemical, ultrastructural, and iron‐handling readouts.

### 2.5 Enhanced Susceptibility to Ferroptosis in Tmsb4x‐CKO Neurons Following mTBI

**Figure 5:**
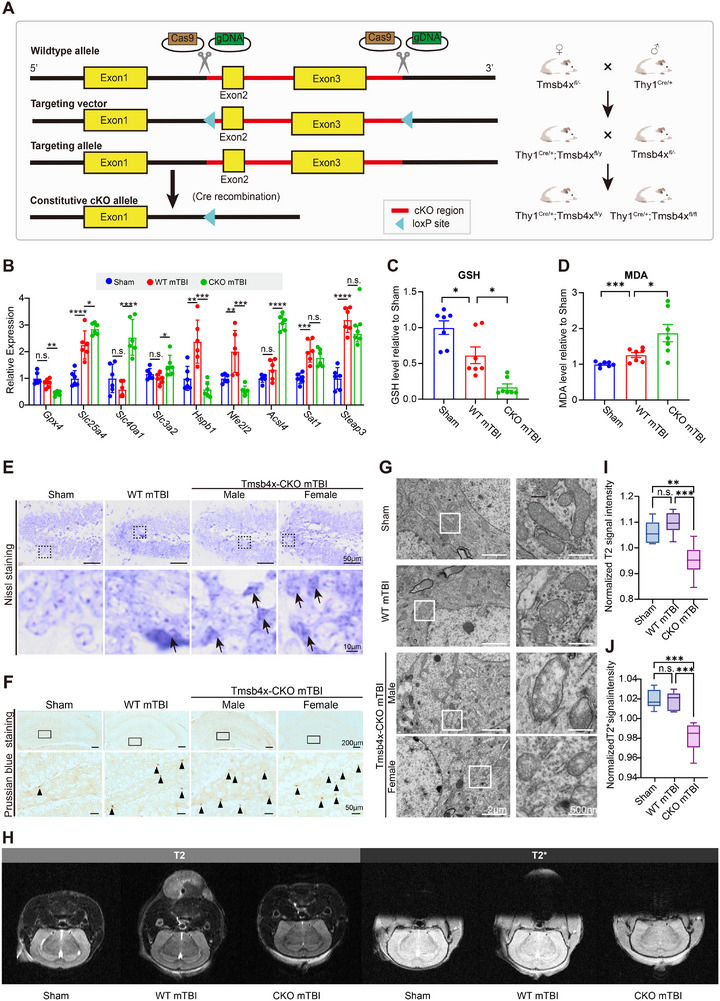
Ferroptosis phenotype in Tmsb4x CKO mice following mTBI. A) Schematic of neuron‐specific Tmsb4x conditional knockout (Thy1^Cre/+^ × Tmsb4x^flox^) and breeding strategy enabling neuronal deletion. B) Relative mRNA levels of ferroptosis‐related genes in sham, mTBI WT, and mTBI CKO hippocampus assessed by qPCR (*n* = 6/group). Shown are mean values ± SEM. *p*‐values measured by two‐way ANOVA with Sidak's multiple comparisons test, *p‐value < 0.05, **p‐value < 0.01, ***p‐value < 0.001, ****p‐value < 0.0001. C) Hippocampal total GSH in sham, mTBI WT, and mTBI CKO (*n* = 7/group). Shown are mean values ± SEM. *p*‐values measured by one‐way ANOVA with Tukey's post hoc test, *p‐value < 0.05. D) Hippocampal MDA in sham, mTBI WT, and mTBI CKO (*n* = 7/group). Shown are mean values ± SEM. *p*‐values measured by one‐way ANOVA with Tukey's post hoc test, *p‐value < 0.05, ***p‐value < 0.001. E) Nissl staining of the hippocampus in mice. F) Perls’ staining of the hippocampus in mice. G) TEM images of the hippocampal DG region, with white boxes highlighting mitochondria. H–J) MRI results and quantitative analysis for mice, assessed using T2 and T2* sequences, where T2 imaging is sensitive to brain water content, and T2* imaging is sensitive to metal ion deposition. Details of the calculation methods are provided in the Methods section (*n* = 3/group). Data are represented as the mean ± SEM. *p*‐values are calculated using one‐way ANOVA with Bonferroni correction, **p‐value < 0.01, ***p‐value < 0.001. Abbreviations: CKO, Conditional Knockout; qPCR, Quantitative Polymerase Chain Reaction; GSH, Glutathione; MDA, Malondialdehyde; MRI, Magnetic Resonance Imaging; TEM, Transmission Electron Microscopy.

To investigate the effects of Tmsb4x deletion on neurons in a mouse model of mTBI, we generated a neuron‐specific conditional knockout by crossing Tmsb4x^flox^ mice with Thy1^Cre/+^ (Tmsb4x‐CKO). Both male (Tmsb4x^flox/y^;Thy1^Cre/+^) and female (Tmsb4x^flox/flox^;Thy1^Cre/+^) CKO littermates were used, with sex‐matched littermate controls lacking Cre. (**Figures** **5**A; S6A, Supporting Information). Though Tmsb4x is located on the sex chromosome, we found that gender did not significantly affect Tmsb4x expression levels under the same treatment conditions (Figure S6B, Supporting Information). We induced mTBI in both WT and Tmsb4x‐CKO mice including both males and females and extracted the hippocampus 48 h post‐injury to assess ferroptosis‐related phenotypes. The results showed significant changes in the expression of ferroptosis‐related genes, such as Gpx4, Slc40a1, Slc3a2, Hspb1, Nfe2l2, and Acsl4, in the hippocampus of Tmsb4x‐CKO mice (Figure 5B), with expression patterns consistent with our in vitro findings. Additionally, the GSH levels in the hippocampus of Tmsb4x‐CKO mice were significantly lower than those in WT mice (Figure 5C), suggesting a reduced antioxidant capacity. Additionally, the MDA levels in the hippocampus of Tmsb4x‐CKO mice were significantly higher than in WT mice, indicating more severe lipid peroxidation (Figure 5D). Neuronal morphological analysis indicated that mTBI caused more severe structural damage in the hippocampal neurons of Tmsb4x‐CKO mice, with blurred neuronal boundaries and dissolution of Nissl bodies (Figure 5E). Prussian blue staining results showed increased iron deposition in the hippocampus of Tmsb4x‐CKO mice (Figure 5F). TEM observations indicated that Tmsb4x‐CKO mice exhibited more severe mitochondrial changes and greater degrees of mitochondrial rupture (Figure 5G). Notably, we also observed reduced water content, increased hemorrhage, and metal ion accumulation in the hippocampus of Tmsb4x‐CKO mice in T2 and T2* MRI sequences (Figures 5H–J; S6C, Supporting Information). In conclusion, these alterations of Tmsb4x‐CKO mice indicate the absence of Tmsb4x exacerbates ferroptosis in hippocampal neurons following mTBI.

### 2.6 Tmsb4x Protects Neurons from Ferroptosis by Regulating Slc2a2

**Figure 6:**
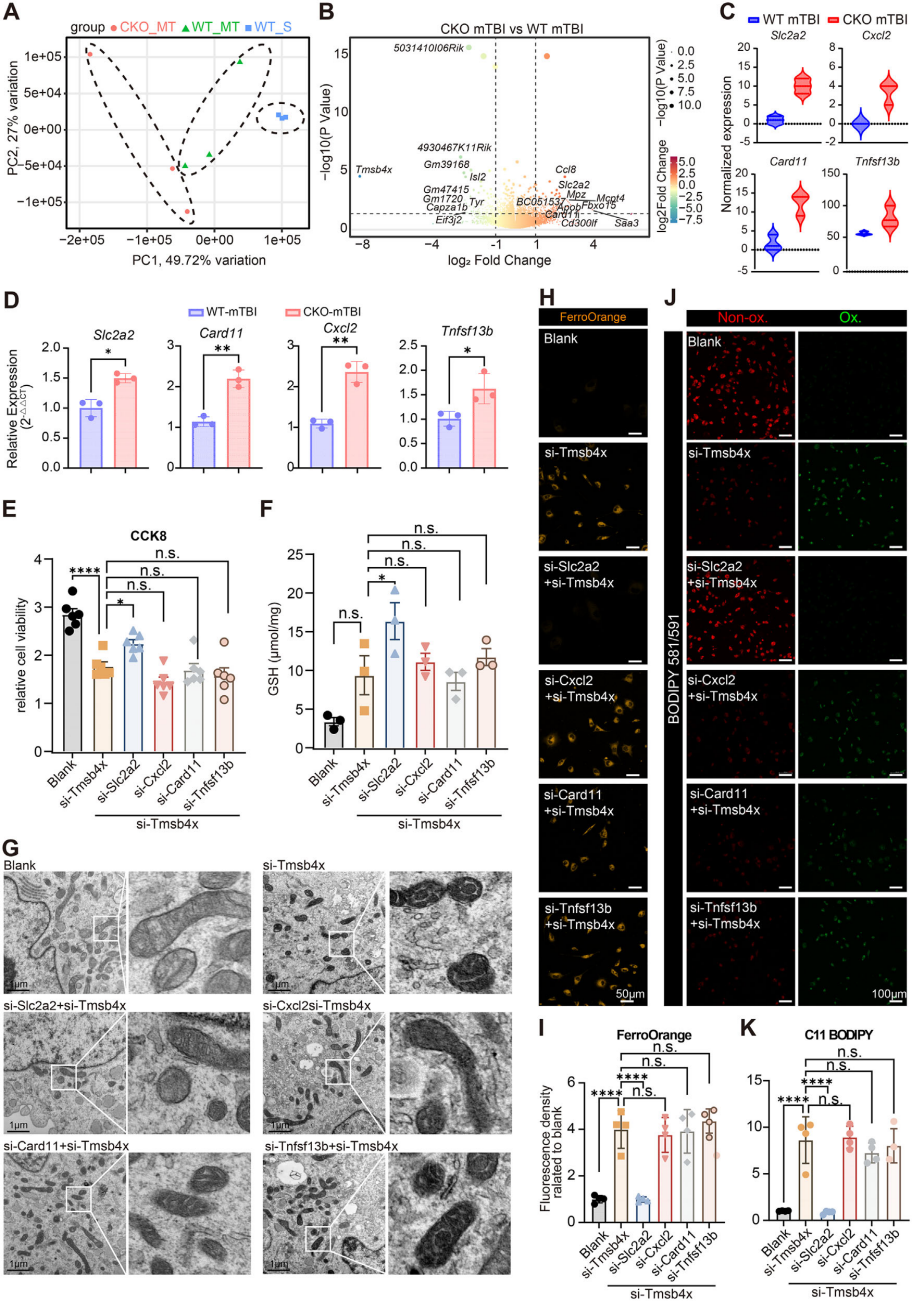
Identification and validation of Slc2a2 as a key gene in exacerbating ferroptosis following Tmsb4x knockdown. A) PCA plot of transcriptional profiles in hippocampal neurons from Tmsb4x‐CKO and WT mice following mTBI, where mice treated with mTBI are labeled “MT”, and those receiving sham treatment are labeled “S”. Each group is represented by color‐coded points, indicating distinct biological replicates and demonstrating transcriptional divergence among groups. B) Volcano plot of DEGs between Tmsb4x‐CKO and WT neurons post‐mTBI, with the top 10 significantly upregulated and downregulated genes labeled, illustrating key gene expression changes associated with ferroptosis. C) Violin plots showing the expression levels of four ferroptosis‐related genes, highlighting gene expression variability and distribution in WT and Tmsb4x‐CKO neurons post‐mTBI. D) RT‐qPCR validation of candidate genes in HT22 cells after Tmsb4x knockdown (*n* = 3/group). Shown are mean values ± SEM from two independent experiments. *p*‐values measured by *t*‐test, *p‐value < 0.05, **p‐value < 0.01. E) Bar plot displaying cell viability of HT22 cells after knockdown (KD) of candidate genes, measured using a CCK8 assay and normalized to the blank group (*n* = 6/group). Data are represented as the mean ± SEM. *p*‐values are calculated using one‐way ANOVA with Bonferroni correction, *p‐value < 0.05, ****p‐value < 0.0001. F) Bar plot showing GSH content in HT22 cells following knockdown of each candidate gene (*n* = 3/group). Shown are mean values ± SEM. *p*‐values measured by one‐way ANOVA with Tukey's post hoc test, *p‐value < 0.05. G) TEM of neuronal mitochondria under the indicated siRNA conditions. Representative fields; insets show enlarged views of boxed areas. Scale bars, as indicated. Images were acquired under identical acquisition settings. H,I) Representative images (H) and quantification (I) of mitochondrial morphology in HT22 cells, stained with MitoTracker, showing low‐magnification views (scale bar = 10 µm) and high‐magnification views (scale bar = 2 µm), demonstrating structural mitochondrial changes following candidate gene knockdown (*n* = 4/group). Shown are mean values ± SEM. *p*‐values measured by one‐way ANOVA with Tukey's post hoc test, ****p‐value < 0.0001. J,K) BODIPY 581/591 staining images (J) and quantification (K) of HT22 cells showing lipid ROS accumulation, where red fluorescence represents the reduced state, and green fluorescence represents the oxidized state (scale bar = 500 µm, *n* = 4/group). Shown are mean values ± SEM. *p*‐values measured by one‐way ANOVA with Tukey's post hoc test, ****p‐value < 0.0001. Abbreviations: PCA, Principal Component Analysis; DEGs, Differentially Expressed Genes; CCK8, Cell Counting Kit‐8; KD, Knockdown; GSH, Glutathione.

We further investigated the protective mechanisms of Tmsb4x against neuronal ferroptosis following mTBI. We generated a neuron‐specific tdTomato red fluorescent protein‐expressing Tmsb4x‐CKO mouse model, Thy‐1^Cre/+^ROSA26^tdTomato/+^ Tmsb4x^f/y^ mice. This model specifically knocks out Tmsb4x in neurons while expressing the tdTomato fluorescent protein (Figure S7A,B, Supporting Information). We subsequently performed FACS to sort hippocampal neurons (Figure S7C,D, Supporting Information) and extracted tdTomato^+^ cells for bulk RNA sequencing. The sequencing revealed specific gene expression changes in the hippocampal neurons of CKO mice following mTBI (**Figures** **6**A,B; S7A,B, Supporting Information). By analyzing DEGs and reviewing existing literature, we identified four ferroptosis‐related genes—*Slc2a2*, *Cxcl2*, *Card11*, and *Tnfsf13b*—that have been reported to be associated with ferroptosis in other studies (Figure 6C). Consistently, RT‐qPCR in HT22 cells showed that si‐Tmsb4x increased the mRNA levels of all four genes (Figure 6D), supporting a transcriptional link between Tmsb4x and this module. Next, we generated Tmsb4x‐KD neurons by transfecting si‐Tmsb4x and co‐transfected siRNAs targeting each of the four genes individually. We assessed a panel of ferroptosis‐related readouts. The Slc2a2‐KD group showed a higher neuronal survival rate (Figure 6E), greater antioxidant capacity (GSH; Figure 6F), more intact mitochondrial ultrastructure (Figures 6G; S9, Supporting Information), lower labile Fe(II) (FerroOrange; Figure 6H,I), and reduced lipid peroxidation (C11‐BODIPY; Figure 6J,K) than the other KD groups. These findings indicate that Slc2a2‐KD can mitigate the increased sensitivity to ferroptosis induced by Tmsb4x‐KD, thereby reducing neuronal damage. Consistent with prior reports that Slc2a2/GLUT2 governs glucose transport and metabolic flux,^[^ ^26^, ^27^ ^]^ we speculate that Tmsb4x upregulation after mTBI may attenuate GLUT2‐mediated glucose efflux, favoring glycolytic flux to generate NADPH and GSH and thereby buffer lipid‐peroxidation‐driven ferroptosis.^[^ ^28^ ^]^

### 2.7 Exogenous Supplementation of Tβ4 Improves Motor and Cognitive Impairments in mTBI Mice

**Figure 7:**
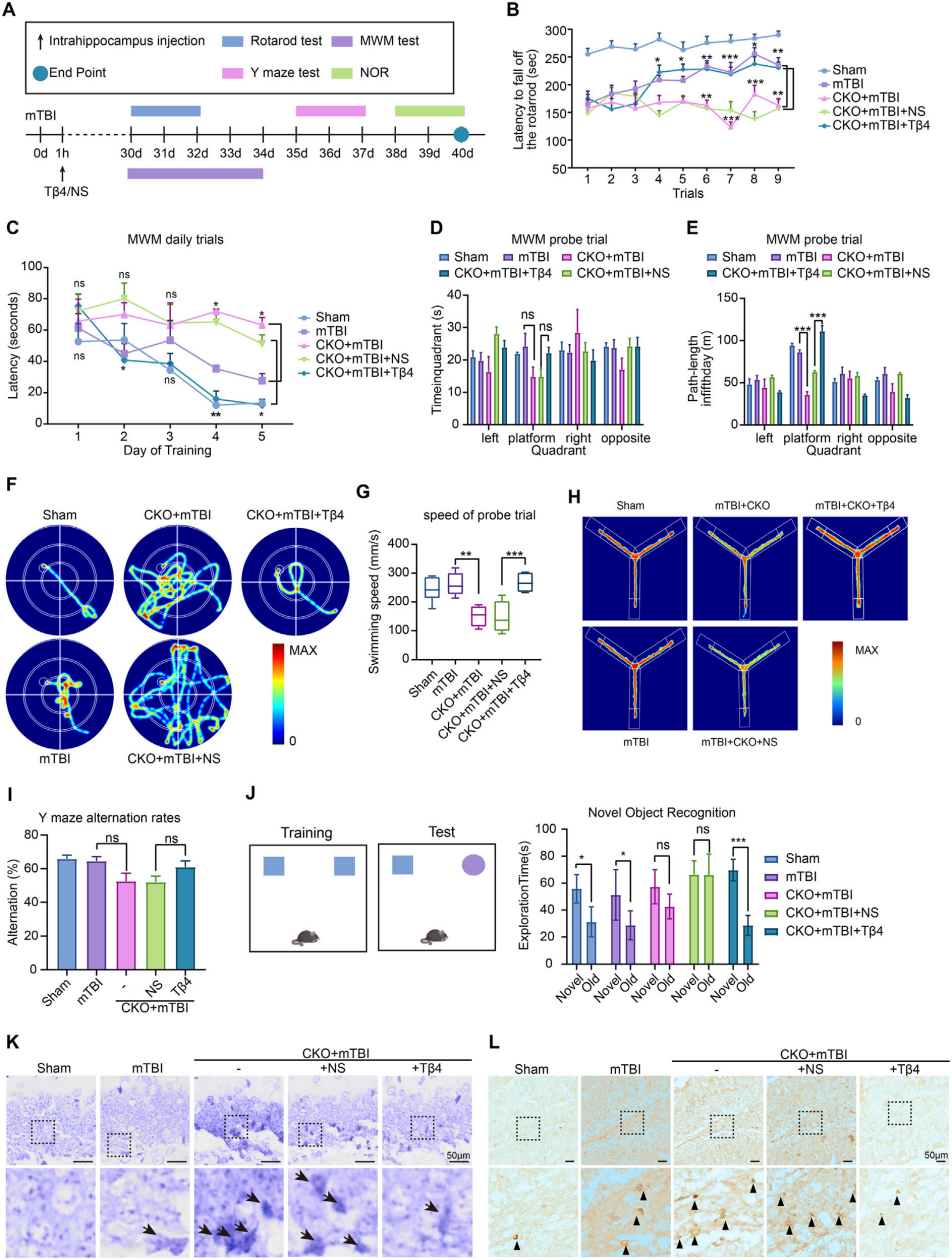
Behavioral assessment demonstrating partial improvement of cognitive and motor deficits in CKO‐mTBI mice following exogenous hippocampal supplementation with Tβ4. A) Schematic of the experimental design, detailing the timeline for intrahippocampal injection, mTBI modeling, and subsequent behavioral tests, including the Rotarod test, MWM test, Y‐maze test, and NOR test. B) Rotarod test results showing the latency to fall for each mouse, with three trials per day over three days, totaling nine trials (*n* = 5/group). Shown are mean values ± SEM. *p*‐values measured by two‐way ANOVA with Sidak's multiple comparisons test, *p‐value < 0.05, **p‐value < 0.01, ***p‐value < 0.001. C) Line graph depicting changes in escape latency across days in the MWM navigation test, illustrating the learning curve over time, with MWM latency data analyzed using two‐way repeated‐measures ANOVA. F) Representative swim path diagrams from the MWM navigation test for each experimental group, showing typical swimming patterns used to locate the platform. D) Bar plot showing the time spent in each quadrant during the MWM probe test. E) Bar plot showing the distance traveled in each quadrant during the MWM probe test. G) Box plot of swimming speed in each quadrant during the MWM probe test (*n* = 5/group). Data are represented as the mean ± SEM. *p*‐values are calculated using one‐way ANOVA with Bonferroni correction, **p‐value < 0.01, ***p‐value < 0.001. H) Heat maps of exploration patterns in the Y‐maze for each group, showing typical movement paths within the maze. I) Bar plot of the percentage alternation rate in the Y‐maze test for each group (*n* = 5/group). Data are represented as the mean ± SEM. *p*‐values are calculated using one‐way ANOVA with Bonferroni correction. J) Schematic diagram of the NOR setup, along with a bar plot showing the time each group spent exploring the novel versus familiar (old) object, where preference for the novel object reflects recognition memory performance. NOR data were analyzed using an unpaired *t*‐test (*n* = 5/group). Shown are mean values ± SEM, *p‐value < 0.05, ***p‐value < 0.001. K) Nissl staining of hippocampal sections from Tmsb4x‐CKO mice post‐mTBI with or without Tβ4 treatment. L) Prussian blue staining of hippocampal sections from Tmsb4x‐CKO mice post‐mTBI with or without Tβ4 treatment. Abbreviations: MWM, Morris Water Maze; NOR, Novel Object Recognition; SEM, Standard Error of the Mean.

Previous experiments have demonstrated that the knockout of Tmsb4x increases the susceptibility of hippocampal neurons to ferroptosis, leading to neuronal loss. Therefore, we aimed to protect the neurons of mTBI mice through the exogenous supplementation of the Tmsb4x protein product, Tβ4, and to investigate its potential to ameliorate the exacerbated motor and cognitive impairments following mTBI. We administered Tβ4 injections into the hippocampus of mice after mTBI and began assessing their motor and cognitive functions one month later (**Figure** **7**A). The results indicated that Tβ4 partially restored motor and balance impairments in CKO mice after injury, as well as somewhat improving learning and memory abilities (Figure 7B–G). Notably, although there was no significant difference in the time spent during the Morris water maze spatial exploration phase between the CKO+mTBI+Tβ4 group and other groups (Figure 7D), the CKO+mTBI+Tβ4 mice traveled a longer path in the target quadrant compared to the saline group (Figure 7E). This increased distance was attributed to their faster swimming speed (Figure 7G), suggesting that their motor abilities had improved. We also found that CKO+mTBI+Tβ4 mice explored longer paths in the Y‐maze experiment, demonstrating increased activity (Figure 7I). Furthermore, the novel object recognition test showed that the mice receiving exogenous Tβ4 displayed greater interest in the new object, spending significantly more time exploring it compared to the old object (Figure 7J). This indicates that Tβ4 injection into the hippocampus can restore object recognition memory in CKO mice. We further investigated the effects of Tmsb4x loss on neuronal integrity and the potential neuroprotective role of Tβ4 following mTBI. Nissl staining of hippocampal sections (Figure 7K) revealed significant neuron loss in the DG region of CKO+mTBI mice, while the treatment with Tβ4 (CKO+mTBI+Tβ4) significantly alleviated these effects, restoring neuronal density and morphology comparable to sham and mTBI controls. Prussian blue staining showed increased DG iron in CKO+mTBI mice, which was reduced by Tβ4 (Figure 7L). Overall, these experimental results suggest that exogenous supplementation of Tβ4 can partially restore motor and cognitive function impairments in mTBI mice. Additionally, Tβ4 treatment alleviates neuronal loss and reduces iron deposition in the DG region, supporting its potential to mitigate ferroptosis‐related damage and provide neuroprotection after mTBI.

## 3 Discussion

mTBI leads to brain tissue damage in two stages. The first stage involves mechanical damage caused by external forces, such as impacts or shock waves, applied to the skull and brain. This results in shear and compression of brain tissue and blood vessels, leading to contusions, lacerations, ruptured blood vessels, and hemorrhaging. Although these changes are often subtle and difficult to observe directly, they can have significant consequences. The second stage encompasses secondary damage that can persist for months or even years, including ischemia, neuroinflammation, alterations in blood‐brain barrier (BBB) permeability, oxidative stress, and neuronal degeneration and death.^[^ ^29^ ^]^ Due to the absence of fractures and significant hemorrhaging in mTBI patients, CT imaging results are often inadequate for diagnosing mTBI.^[^ ^30^ ^]^ Furthermore, the diagnosis of mTBI lacks relatively objective and effective biological evidence. Neuronal degeneration and death in the hippocampus following mTBI are critical factors contributing to cognitive dysfunction. Therefore, understanding the mechanisms of neuronal death and the associated changes in transcriptional activity is essential for developing effective therapeutic targets to mitigate secondary cognitive impairments after mTBI. The single‐cell transcriptomic and epigenetic maps of the hippocampus in mTBI mice constructed in this study, along with the gene sets related to cell death, provide valuable resources for future research into mTBI and the mechanisms underlying hippocampal neuronal loss.

Neuronal death can occur through various mechanisms, including necrosis that rapidly follows injury and programmed cell death.^[^ ^31^ ^]^ Most studies on neuronal death following mTBI have focused on apoptosis. For instance, Renana Baratz et al. found that the apoptotic marker BID is significantly upregulated 72 h post‐mTBI, and immediate treatment with 3,6'‐dithiothalidomide can significantly improve neuronal apoptosis and cognitive impairments associated with mTBI.^[^ ^32^ ^]^ However, recent discoveries have identified various novel forms of cell death, such as ferroptosis, characterized by iron overload and lipid peroxidation; cuproptosis, induced by copper ion toxicity; and pyroptosis, mediated by GSDMD‐induced pore formation. Additionally, more complex forms of cell death, such as necroptosis, which exhibit features of both necrosis and apoptosis, and pan‐apoptosis, characterized by elements of apoptosis and necroptosis, have also been identified. These different modes of cell death are interregulated and collectively influence cellular fate.^[^ ^33^ ^]^ In our study, we utilized an established gene set for various forms of cell death, along with published cell death‐specific genes, to construct gene sets for 14 different cell death pathways, allowing us to assess their activity in neuronal death. We found that the activation levels of these cell death‐specific genes varied among different subtypes of hippocampal neurons, with ferroptosis being the most extensively and significantly activated. While ferroptosis has been implicated in hippocampal pathology after TBI, our data extend this literature by resolving subtype‐specific activation patterns at single‐nucleus resolution.^[^ ^15^ ^]^ Therefore, our analysis focused on transcriptomic changes associated with ferroptosis, including transcriptional regulatory features of mitochondrial damage and key regulatory genes involved in neuronal ferroptosis. Notably, we discovered that c‐Jun‐mediated transcriptional regulation of Tmsb4x plays a critical role in neuronal ferroptosis in the hippocampus. Furthermore, exogenous supplementation with Tβ4 helps restore learning and memory functions in mTBI mice, providing important clinical intervention insights for mTBI.

Within our neuronal subtype clustering, we identified two cell populations that could not be characterized by known hippocampal neuronal subtypes: Neuronal subtype 1 and Neuronal subtype 2. Neuronal subtype 1 exhibited high expression of genes such as Tshz2 and Arpp21, resembling the “Neuronal Subtype 1” identified in single‐cell sequencing of mouse hippocampus by Douglas Arneson and colleagues. Their analysis suggested that this cell group has differentiation and self‐renewal potential. Our findings indicated that this group expresses several genes associated with neuronal development and synaptogenesis, such as Nrxn1 and Cntnap4. GO analysis revealed that the characteristic genes of Neuronal subtype 1 are primarily involved in pathways related to dendrite development, actin filament organization, protein localization to the cell periphery, actin filament bundle assembly, and actin filament bundle organization (Figure S3C, Supporting Information). These pathways are crucial stages in the development and branching of immature neurons, suggesting that this population consists of neurons still undergoing development. In contrast, Neuronal subtype 2 expressed genes related to neuronal migration and axon guidance, such as Tenm2 and Nrg1, indicating that these neurons may be migrating within the hippocampus.

In our in vivo experiments examining the relationship between conditional knockout of Tmsb4x and neuronal ferroptosis in the hippocampus, we employed the Cre‐LoxP system to construct conditional knockout mice. Given that Tmsb4x is X‐linked, sex may influence its expression. To explore this, we assessed TMSB4X protein levels in the hippocampus of male and female mice. Our results showed no significant sex differences in TMSB4X protein expression in either the sham or mTBI groups (Figure S6B, Supporting Information). This phenomenon can be attributed to X‐chromosome inactivation in female mammals, wherein one of the two X chromosomes undergoes heterochromatinization and becomes inactive, ensuring that gene expression products on sex chromosomes remain largely consistent between males and females.

Tβ4 is a naturally occurring protein in mammals with various biological functions. Clinically, Tβ4 is primarily used to promote wound healing, tissue regeneration, and anti‐inflammatory effects. Recent studies in neuroscience have demonstrated the protective role of Tβ4 against neuronal degeneration. For example, Patrizia Popoli et al. found that Tβ4 protects neurons in models of excitotoxicity induced by glutamate or kainate,^[^ ^34^ ^]^ while Hao Yang et al. showed that Tβ4 promotes neuronal survival and neurogenesis by enhancing the expression of L1.^[^ ^35^ ^]^ In 2022, Joanna I. Lachowicz first reported that Tβ4 acts as an endogenous iron chelator, inhibiting macrophage ferroptosis and increasing the expression of oxidative stress‐related genes.^[^ ^23^ ^]^ However, the role of Tβ4 in neuronal ferroptosis has not yet been reported. Our study reveals that the knockdown of Tmsb4x expression increases the susceptibility of cells to ferroptosis, a process regulated by changes in c‐Jun transcription factor activity, which subsequently affects Slc2a2 and contributes to neuronal ferroptosis. Tmsb4x‐CKO mice exhibit more severe neuronal ferroptosis and greater impairments in motor and cognitive functions following mTBI, while exogenous supplementation with Tβ4 reverses these changes. This indicates that Tβ4 has a protective role in neuronal ferroptosis, alleviating neuronal loss after mTBI and preserving the integrity of neural circuits and hippocampal function. This finding offers valuable intervention strategies for clinical treatment of cognitive and motor dysfunction related to hippocampal damage after mTBI.

As a water‐soluble molecule, Tβ4 lacks the ability to passively diffuse across cell membranes. Instead, it enters cells through endocytosis or other active transport mechanisms by binding to receptors or transport proteins on the cell surface. Therefore, for recombinant protein formulations to be effectively applied in human health, it is necessary to conjugate Tβ4 with lipophilic groups or incorporate it into nanoscale delivery systems to enhance its cellular uptake.

In this study, we successfully constructed comprehensive single‐cell transcriptomic and epigenomic atlas of the hippocampus in the mTBI mice. Based on a self‐established gene set, we discovered distinct fate‐determining patterns of different hippocampal neuron subtypes, with the ferroptosis pathway being widely and significantly activated. Further mechanistic investigations revealed that the c‐Jun‐regulated gene Tmsb4x is upregulated in both mouse and human hippocampal tissues, where it plays a protective role against neuronal ferroptosis. This gene enhances the cell's antioxidant capacity by regulating the energy metabolism‐related gene Slc2a2. Exogenous supplementation with the recombinant protein Tβ4, a product of Tmsb4x, significantly reduced cognitive and motor function impairments in mTBI mice. These findings are crucial for understanding the pathological processes underlying neuronal loss after mTBI and hold promise for identifying new targets for the treatment of mTBI.

## 4 Experimental Section

### Study Design

Hippocampal single‐nucleus library preparation and sequencing were performed using hippocampal samples from mice in the mTBI (*n* = 3) and sham (*n* = 3) groups. ATAC‐seq library preparation and sequencing were conducted on hippocampal samples from the mTBI (*n* = 3) and sham (*n* = 3) groups. Bulk RNA‐seq library preparation and sequencing were performed using nuclear samples from three groups of mice: CKO + mTBI (*n* = 3), WT + mTBI (*n* = 3), and WT + sham (*n* = 3). The number of samples and grouping for both in vivo and in vitro experiments were detailed in the corresponding methodological sections or indicated in the relevant figures. To minimize bias, randomization was applied during group assignment, sample processing, and data collection. Mice were randomly assigned to experimental groups before the induction of mTBI or sham procedures. Investigators responsible for data collection, library preparation, sequencing, and analysis were blinded to group allocations. Data analysts were provided with anonymized sample identifiers to ensure an unbiased interpretation of the results.

### Animals

This study used adult male and female C57BL/6 mice (15–25 g body weight, 8–15 weeks old) or transgenic mice. C57BL/6J‐Tmsb4x^em1(flox)Cya^ mice were purchased from Cyagen (strain number: CKOCMP‐19241‐Tmsb4x‐B6J‐VA). These mice were generated using CRISPR/Cas9‐mediated gene editing, targeting exons 2 and 3 of TMSB4X for conditional deletion. Two distinct CRISPR gRNAs (gR#1 and gR#2) were designed to target different exon regions of TMSB4X, leading to the successful generation of mice carrying the flox‐Tmsb4x allele. The final conditional knockout female mice were designated as Tmsb4x^flox+/−^. Thy1‐Cre (FVB/N‐Tg(Thy1‐cre)1Vln/J) mice were obtained from Cyagen (stock number: C001306), while the Ai9 transgenic strain (JAX 007909) was acquired from The Jackson Laboratory. All animal housing, maintenance, surgical procedures, post‐experimental monitoring, and behavioral assessments were conducted in accordance with the NIH guidelines for the care and use of laboratory animals. Mice were housed in a controlled environment with a temperature of 19–22 °C, relative humidity of 45–55%, and a 12‐h light/dark cycle. Food and water were provided ad libitum. All animal experiments were approved by the Ethics Committee of Sichuan University (Approval No. K2021024) and conducted under its guidelines for the ethical use of animals in research.

### Human Tissue Samples and Ethics Statement

Human tissue samples were obtained from the Chengdu Public Security Bureau in compliance with the ethical principles outlined in the Declaration of Helsinki. The collection and use of human samples were approved by the Ethics Committee of West China Hospital, Sichuan University (Approval No. 2022‐153). Hippocampal samples from individuals with traumatic brain injury (TBI) were obtained from deceased individuals diagnosed with TBI (TBI *n* = 3 / Control *n* = 3). Postmortem examination revealed bilateral temporal muscle hemorrhage, extensive galeal hematoma, subdural hematoma, and subarachnoid hemorrhage. Negative control hippocampal samples were collected from deceased individuals who died from non‐TBI‐related diseases.

### mTBI Modeling and mNSS Scoring

To induce anesthesia, an inhalation anesthesia system (RWD Life Science) was used to vaporize isoflurane, enabling rapid induction and recovery. Mice were anesthetized with 4% isoflurane for induction, followed by 1.5% isoflurane for maintenance for 90 s or until the completion of the procedure and suturing. During surgery, mice were placed in a prone position with their heads positioned within an anesthesia mask. A sponge pad was placed beneath the ventral side of the mice to absorb impact forces and prevent skull fractures caused by the injury. mTBI was induced using an electromagnetic‐controlled cortical impactor (eCCI‐6.3, Custom Design & Fabrication, Inc.), following the protocol described by Yang Q. et al.^[^ ^36^ ^]^ The impact parameters were as follows: impactor tip diameter: 3 mm, impact velocity: 3.5 m s^−1^, impact depth: 1 mm, and dwell time: 150 ms. One hour after injury, the modified Neurological Severity Score (mNSS) was used to assess the severity of brain injury. Mice with mNSS scores of 3–4 were considered to have successfully undergone mTBI modeling.^[^ ^37^ ^]^ The mNSS scoring criteria and assessment protocol were based on the method described by Flierl et al.^[^ ^38^ ^]^ Mice in the sham group underwent anesthesia and scalp suturing but did not receive an impact injury.

### snRNA‐Seq Library Preparation and Sequencing

Hippocampal single‐nucleus RNA sequencing (snRNA‐seq) was performed on hippocampal samples obtained from male mice in the mTBI (*n* = 3) and sham (*n* = 3) groups. Tissue collection was conducted 48 h post‐injury, following cardiac perfusion with saline under anesthesia. The brain was rapidly extracted, and the hippocampus was dissected in ice‐cold PBS. Single‐nucleus isolation and preparation were performed following the protocol described by Yang Q et al.^[^ ^36^ ^]^ Nuclei were counted using trypan blue staining, and the concentration was adjusted to 1000 nuclei / µL. 10× Genomics library preparation and single‐nucleus RNA sequencing were conducted by NovelBio Co., Ltd. (Shanghai, China). Libraries were generated using the 10× Genomics Chromium Controller Instrument and Chromium Single Cell 3' v3 Reagent Kits (10× Genomics, Pleasanton, USA). Sequencing was performed on the NovaSeq 6000 platform (Illumina, USA), following the 2 × 150 bp paired‐end sequencing protocol, as referenced in Li M. et al.^[^ ^39^ ^]^

### ATAC‐Seq Library Preparation and Sequencing

Hippocampal samples from mTBI (*n* = 3) and sham (*n* = 3) group mice were used for ATAC‐seq library preparation and sequencing. The library construction and sequencing procedures followed the previously established experimental protocol.^[^ ^40^ ^]^ Raw reads were processed as detailed in ATAC‐seq Data Processing.

### Bulk RNA‐Seq Library Preparation and Sequencing

Bulk RNA sequencing was performed using cell suspensions obtained after fluorescence‐activated cell sorting (FACS) (*n* = 3 per group). Library preparation and sequencing procedures were conducted according to the previously established experimental protocol.^[^ ^41^ ^]^

### Cell‐Type Proportion Analysis

Cell‐type proportions were analyzed by first exporting the number of cells for each cell type using the table function. The prop.table function was then used to calculate the proportion of each cell type. Finally, a bar plot was generated to visualize the cell‐type proportions across different conditions.

### ATAC‐Seq Data Processing

The processing of ATAC‐seq sequencing data, including differential peak analysis, motif analysis, enrichment of genomic signals around transcription start sites (TSS), and peak visualization, was performed following the methods described in Li M et al.^[^ ^40^ ^]^

### qPCR Analysis

Quantitative PCR (qPCR) was performed following the previously used experimental protocol.^[^ ^42^ ^]^ Primer sequences for target genes are provided in Table S8 (Supporting Information).

### Ferroptosis Induction

Ferroptosis was induced using the ferroptosis activator RSL3 (Selleck, Cat# S8155). Prior to the experiment, RSL3 was dissolved in DMSO to prepare a stock solution, which was further diluted to the desired working concentration. HT22 cells were treated with 3 µm RSL3 at 37 °C for 24 h, while the control group received an equivalent volume of DMSO to account for solvent effects.

### Ferroptosis Inhibition

For pharmacological inhibition of ferroptosis, cells were treated with Ferrostatin‐1 (Fer‐1, Selleck, #S7243) dissolved in DMSO. Fer‐1 was added at the time of RSL3 addition at a final concentration of 1 µm, with vehicle volumes matched across conditions.

### Ferrous Ion (Fe^2^⁺) Detection

Ferrous ion levels in HT22 cells were detected using FerroOrange (Dojindo, Cat# F374). A 1 mM stock solution was prepared as per the manufacturer's recommendations and diluted 1:1000 in PBS to create the working solution. Cells were incubated in the FerroOrange working solution at 37 °C for 30 min. After staining, cells were gently washed 2–3 times with pre‐warmed (37 °C) PBS, and fluorescence imaging was performed using a Zeiss LSM 810 confocal microscope under the Cy3 channel.

### Morris Water Maze (MWM) Test

The MWM test was conducted to assess cognitive function in mice (*n* = 5), following previously established protocols.^[^ ^43^ ^]^ The maze consisted of a large circular pool filled with opaque water, concealing a submerged escape platform. The pool was divided into four quadrants. Mice underwent four trials per day to evaluate their ability to locate the hidden platform. Each trial began from one of four designated starting points, with mice being gently placed in the water facing the wall. Mice were given a maximum of 90 s to locate the submerged platform. If a mouse failed to find the platform within this time, it was manually guided onto the platform and allowed to remain for 10 s to familiarize itself with the location. A minimum 4‐min inter‐trial interval was maintained to allow for recovery between consecutive trials. On Day 5, a probe trial was conducted, in which the platform was removed, and the swimming path, the number of entries into the target quadrant (where the platform was previously located), and the time spent in the target quadrant were recorded over 90 s. The primary outcome measures included the latency to find the platform across trials and the time spent in the target quadrant and the number of entries into the previous platform location on Day 5. Shorter latencies and increased time spent in the target quadrant indicated improved spatial learning and long‐term memory, reflecting enhanced hippocampus‐dependent cognitive function.

### Rotarod Test

The rotarod apparatus consisted of a rotating rod and an infrared recording system. The testing area was dimly lit to minimize mouse anxiety and ensure optimal visibility. Before testing, each mouse underwent an acclimation period to reduce stress‐related variability. On Day 1, mice were individually placed on a stationary rod for 5 min, allowing them to familiarize themselves with the rotarod and testing environment without rotational stress. The test was conducted over three consecutive days. At the beginning of each trial, the rod rotated at 4 rpm and gradually accelerated to 40 rpm over 5 min. Mice were placed on the rod, and the latency to fall (i.e., time before falling off) was recorded. To assess motor coordination and learning progression, each mouse underwent three trials per day, with 15‐min intervals between trials to prevent fatigue. If a mouse clung to the rod and remained stable for the full 5‐min duration, the trial was automatically terminated. Performance on the rotarod test was monitored using an automated tracking system, and fall latency was recorded for each mouse. Videos were captured and analyzed using the BAS‐100 Animal Behavior Analysis System (Techman Co., Shanghai, China).

### Y‐Maze Test

The Y‐maze consists of three arms positioned at 120‐degree angles and was used to assess spatial working memory in mice. All arms were of equal dimensions to ensure experimental consistency. The maze was placed in a well‐lit and quiet room to minimize external disturbances. On the testing day, each mouse was placed at the end of a designated start arm and allowed to freely explore the maze for 5 min. The entire process was recorded using an overhead camera connected to a tracking system. Alternation behavior was defined as consecutive entries into all three arms (e.g., ABC, CAB, BCA) without repetition, reflecting memory capacity and exploratory curiosity toward novel areas. The alternation rate was calculated using the formula: (alternation times / (total entry times – 2)) × 100%.

### Novel Object Recognition (NOR) Test

NOR test was performed following the previously established experimental protocol.^[^ ^44^ ^]^

### Statistical Analysis

Statistical analyses were performed using GraphPad Prism version 10.1.2 (GraphPad Software, San Diego, CA, USA), and data were presented as means ± SEM. qPCR data were analyzed using the 2‐ΔΔCt method, with target gene expression normalized to the reference gene and expressed as fold change relative to the control group. For comparisons between experimental groups, Student's *t*‐test was used for two‐group comparisons, while the one‐way ANOVA test followed by Tukey's post hoc test was applied for comparisons involving more than two groups, with Bonferroni correction used for multiple comparisons. For behavioral assays, MWM latency data were analyzed using two‐way repeated‐measures ANOVA to assess the interaction of different factors over time, while Novel Object Recognition (NOR) data were analyzed using an unpaired t‐test to compare the time spent exploring the novel object versus the familiar object. The exact *n* values (biological replicates for in vitro assays or animals per group for in vivo studies) are indicated in the figure legends. All statistical analyses were performed using R (version 4.0.2) or GraphPad Prism (version 10.1.2), and statistical significance was denoted as follows: *p < 0.05, **p < 0.01, ***p < 0.001, ****p < 0.0001, n.s. not significant.

## Conflict of Interest

The authors declare no conflict of interest.

## Ethics Statement

All animal experiments were approved by the Ethics Committee of Sichuan University (Approval No. K2021024) and conducted under its guidelines for the ethical use of animals in research. Human tissue samples were obtained from the Chengdu Public Security Bureau in compliance with the ethical principles outlined in the Declaration of Helsinki. The collection and use of human samples were approved by the Ethics Committee of West China Hospital, Sichuan University (Approval No. 2022‐153).

## Author Contributions

M.L., Q.Y., and S.Q. contributed equally to this work. M.L. and Q.Y. were responsible for conceptualization. S.Q. and Y.X. carried out methodology. M.L. and Q.Y. performed the investigation. Y.C. and X.L. handled visualization. M.L., Y.S., Y.C., and Y.S. conducted data analysis. M.L., Z.Y., W.L., X.C., and L.Z. provided supervision. X.C. acquired funding.

## Supporting information



Supporting Information

Supplemental Data File

Supplemental Table Legends

Supplemental Table 1

Supplemental Table 2

Supplemental Table 3

Supplemental Table 4

Supplemental Table 5

Supplemental Table 6

Supplemental Table 7

Supplemental Table 8

## Data Availability

The data that support the findings of this study are openly available in Gene Expression Omnibus at https://www.ncbi.nlm.nih.gov/geo/query/acc.cgi?acc = GSE292126; https://www.ncbi.nlm.nih.gov/geo/query/acc.cgi?acc = GSE292127; https://www.ncbi.nlm.nih.gov/geo/query/acc.cgi?acc = GSE292715, reference number 292715.

## References

[1] M. Faul , L. Xu , M. Wald , V. Coronado , A. M. Dellinger , Inj. Prev. 2010, 16, A268.

[2] H. M. Bramlett , W. D. Dietrich , J. Neurotrauma 2015, 32, 1834. 25158206 10.1089/neu.2014.3352 PMC4677116

[3] D. I. Katz , S. I. Cohen , M. P. Alexander , Handb. Clin. Neurol. 2015, 127, 131. 25702214 10.1016/B978-0-444-52892-6.00009-X

[4] F. Y. Kwok , T. M. Lee , C. H. Leung , W. S. Poon , Brain Inj. 2008, 22, 740. 18787983 10.1080/02699050802336989

[5] T. W. McAllister , Dialogues Clin. Neurosci. 2011, 13, 287. 22033563 10.31887/DCNS.2011.13.2/tmcallister PMC3182015

[6] L. B. Tucker , A. H. Fu , J. T. McCabe , J. Neurotrauma 2021, 38, 1585. 33622092 10.1089/neu.2021.0025 PMC8126427

[7] K. S. Anand , V. Dhikav , Ann. Indian Acad. Neurol. 2012, 15, 239. 23349586 10.4103/0972-2327.104323 PMC3548359

[8] F. Girgis , J. Pace , J. Sweet , J. P. Miller , Front. Syst. Neurosci. 2016, 10, 8. 26903824 10.3389/fnsys.2016.00008 PMC4746250

[9] H. Zhou , L. Chen , X. Gao , B. Luo , J. Chen , J. Neuropathol. Exp. Neurol. 2012, 71, 348. 22437344 10.1097/NEN.0b013e31824ea078 PMC3311037

[10] R. Raghupathi , Brain Pathol. 2004, 14, 215. 15193035 10.1111/j.1750-3639.2004.tb00056.x PMC8096005

[11] Z. Tian , Z. Cao , E. Yang , J. Li , D. Liao , F. Wang , T. Wang , Z. Zhang , H. Zhang , X. Jiang , X. Li , P. Luo , Neural Regen. Res. 2023, 18, 2711. 37449635 10.4103/1673-5374.374654 PMC10358661

[12] P. Tsvetkov , S. Coy , B. Petrova , M. Dreishpoon , A. Verma , M. Abdusamad , J. Rossen , L. Joesch‐Cohen , R. Humeidi , R. D. Spangler , J. K. Eaton , E. Frenkel , M. Kocak , S. M. Corsello , S. Lutsenko , N. Kanarek , S. Santagata , T. R. Golub , Science 2022, 375, 1254. 35298263 10.1126/science.abf0529 PMC9273333

[13] B. R. Stockwell , J. P. Friedmann Angeli , H. Bayir , A. I. Bush , M. Conrad , S. J. Dixon , S. Fulda , S. Gascón , S. K. Hatzios , V. E. Kagan , K. Noel , X. Jiang , A. Linkermann , M. E. Murphy , M. Overholtzer , A. Oyagi , G. C. Pagnussat , J. Park , Q. Ran , C. S. Rosenfeld , K. Salnikow , D. Tang , F. M. Torti , S. V. Torti , S. Toyokuni , K. A. Woerpel , D. D. Zhang , Cell 2017, 171, 273. 28985560 10.1016/j.cell.2017.09.021 PMC5685180

[14] L. Galluzzi , I. Vitale , S. A. Aaronson , J. M. Abrams , D. Adam , P. Agostinis , E. S. Alnemri , L. Altucci , I. Amelio , D. W. Andrews , M. Annicchiarico‐Petruzzelli , A. V. Antonov , E. Arama , E. H. Baehrecke , N. A. Barlev , N. G. Bazan , F. Bernassola , M. J. M. Bertrand , K. Bianchi , M. V. Blagosklonny , K. Blomgren , C. Borner , P. Boya , C. Brenner , M. Campanella , E. Candi , D. Carmona‐Gutierrez , F. Cecconi , F. K.‐M. Chan , N. S. Chandel , et al., Cell Death Differ. 2018, 25, 486. 29362479 10.1038/s41418-017-0012-4 PMC5864239

[15] D. Wang , S. Zhang , X. Ge , Z. Yin , M. Li , M. Guo , T. Hu , Z. Han , X. Kong , D. Li , J. Zhao , L. Wang , Q. Liu , F. Chen , P. Lei , J. Neuroinflammation 2022, 19, 185. 35836233 10.1186/s12974-022-02550-7 PMC9281149

[16] M. Gao , J. Yi , J. Zhu , A. M. Minikes , P. Monian , C. B. Thompson , X. Jiang , Mol. Cell 2019, 73, 354. 30581146 10.1016/j.molcel.2018.10.042 PMC6338496

[17] S. Oura , S. Kazi , A. Savolainen , K. Nozawa , J. Castaneda , Z. Yu , H. Miyata , R. M. Matzuk , J. N. Hansen , D. Wachten , M. M. Matzuk , R. Prunskaite‐Hyyryläinen , PLoS Genet. 2020, 16, 1008954. 10.1371/journal.pgen.1008954 PMC7444823 32785227

[18] B. Li , F. Liu , X. Chen , T. Chen , J. Zhang , Y. Liu , Y. Yao , W. Hu , M. Zhang , B. Wang , L. Liu , K. Chen , Y. Wu , Circulation 2024, 149, 1268. 38362779 10.1161/CIRCULATIONAHA.123.064489 PMC11017836

[19] S. A. Predescu , D. N. Predescu , I. Knezevic , I. K. Klein , M. ABJJobc , J. Biol. Chem. 2007, 282, 17166. 17405881 10.1074/jbc.M608996200

[20] Y. Li , Y. Liu , K. Jin , R. Dong , C. Gao , L. Si , Z. Feng , H. Zhang , H. Tian , Front. Cell Dev. Biol. 2021, 9, 771824. 34926459 10.3389/fcell.2021.771824 PMC8672271

[21] C. Dunn , C. Wiltshire , A. MacLaren , G. DAJCs , Cell. Signal. 2002, 14, 585. 11955951 10.1016/s0898-6568(01)00275-3

[22] L. Didon , R. K. Zwick , I. W. Chao , M. S. Walters , R. Wang , N. R. Hackett , R. G. Crystal , Respir. Res. 2013, 14, 70. 23822649 10.1186/1465-9921-14-70 PMC3710277

[23] J. I. Lachowicz , G. Pichiri , M. Piludu , S. Fais , G. Orrù , T. Congiu , M. Piras , G. Faa , D. Fanni , G. Dalla Torre , X. Lopez , K. Chandra , K. Szczepski , L. Jaremko , M. Ghosh , A.‐H. Emwas , M. Castagnola , M. Jaremko , E. Hannappel , P. Coni , Int. J. Mol. Sci. 2022, 23, 551. 35008976 10.3390/ijms23010551 PMC8745404

[24] M. A. Evans , N. Smart , K. N. Dubé , S. Bollini , J. E. Clark , H. G. Evans , L. S. Taams , R. Richardson , M. Lévesque , P. Martin , K. Mills , J. Riegler , A. N. Price , M. F. Lythgoe , P. R. Riley , Nat. Commun. 2013, 4, 2081. 23820300 10.1038/ncomms3081 PMC3797509

[25] J. Zhu , L.‐P. Su , L. Ye , K.‐O. Lee , J.‐H. Ma , Diabetes Res. Clin. Pract. 2012, 96, 53. 22217673 10.1016/j.diabres.2011.12.009

[26] A. Michau , G. Guillemain , A. Grosfeld , S. Vuillaumier‐Barrot , T. Grand , M. Keck , S. L'Hoste , D. Chateau , P. Serradas , J. Teulon , P. De Lonlay , R. Scharfmann , E. Brot‐Laroche , A. Leturque , M. Le Gall , J. Biol. Chem. 2013, 288, 31080. 23986439 10.1074/jbc.M113.469189 PMC3829421

[27] L. J. McCulloch , M. van de Bunt , M. Braun , K. N. Frayn , A. Clark , A. L. Gloyn , Mol. Genet. Metab. 2011, 104, 648. 21920790 10.1016/j.ymgme.2011.08.026

[28] L. Plecitá‐Hlavatá , M. Jabůrek , B. Holendová , J. Tauber , V. Pavluch , Z. Berková , M. Cahová , K. Schröder , R. P. Brandes , D. Siemen , P. Ježek , Diabetes 2020, 69, 1341. 32245800 10.2337/db19-1130

[29] S. Y. Ng , A. Y. W. Lee , Front. Cell. Neurosci. 2019, 13, 528. 31827423 10.3389/fncel.2019.00528 PMC6890857

[30] L. L. Fernandez , D. P. Griswold , B. Fariyike , S. Aristizabal , B. Perez , A. M. Rubino , J. Surg. Protoc. Res. Methodol. 2022, 2022, snab010.

[31] E. Hendriksen , D. van Bergeijk , R. S. Oosting , F. A. J. N. Redegeld , Neurosci. Biobehav. Rev. 2017, 79, 119. 28499503 10.1016/j.neubiorev.2017.05.001

[32] R. Baratz , D. Tweedie , J.‐Y. Wang , V. Rubovitch , W. Luo , B. J. Hoffer , N. H. Greig , C. G. Pick , J. Neuroinflammation 2015, 12, 45. 25879458 10.1186/s12974-015-0237-4 PMC4352276

[33] D. Moujalled , A. Strasser , J. R. Liddell , Cell Death Differ. 2021, 28, 2029. 34099897 10.1038/s41418-021-00814-y PMC8257776

[34] P. Popoli , R. Pepponi , A. Martire , M. Armida , A. Pèzzola , M. Galluzzo , M. R. Domenici , R. L. Potenza , M. T. Tebano , C. Mollinari , D. Merlo , E. Garaci , Ann. N. Y. Acad. Sci. 2007, 1112, 219. 17947590 10.1196/annals.1415.033

[35] H. Yang , X. Cheng , Q. Yao , J. Li , G. Ju , Neurochem. Res. 2008, 33, 2269. 18461449 10.1007/s11064-008-9712-y

[36] Q. Yang , L. Zhang , M. Li , Y. Xu , X. Chen , R. Yuan , X. Ou , M. He , M. Liao , L. Zhang , H. Dai , M. Lv , X. Xie , W. Liang , X. Chen , Genome Res. 2023, 33, 1818. 37730437 10.1101/gr.277881.123 PMC10691476

[37] N. Henninger , J. Bouley , E. M. Sikoglu , J. An , C. M. Moore , J. A. King , R. Bowser , M. R. Freeman , R. H. Brown , Brain 2016, 139, 1094. 26912636 10.1093/brain/aww001 PMC5006226

[38] M. A. Flierl , P. F. Stahel , K. M. Beauchamp , S. J. Morgan , W. R. Smith , E. Shohami , Nat. Protoc. 2009, 4, 1328. 19713954 10.1038/nprot.2009.148

[39] M. Li , Y. Jin , Y. Xu , Y. Sun , R. Yuan , X. Zhang , S. Qu , M. Lv , M. Liao , W. Liang , L. Zhang , X. Chen , Int. J. Legal Med. 2024, 138, 1273. 38491322 10.1007/s00414-024-03206-2

[40] M. Li , X. Chen , Q. Yang , S. Cao , S. Wyler , R. Yuan , L. Zhang , M. Liao , M. Lv , F. Wang , Y. Guo , J. Zhou , L. Zhang , X. Xie , W. Liang , Sci. Data 2023, 10, 670. 37783673 10.1038/s41597-023-02563-8 PMC10545782

[41] Y.‐J. Jiang , S.‐Q. Cao , L.‐B. Gao , Y.‐Y. Wang , B. Zhou , X. Hu , Y. Pu , Z.‐L. Li , Q. Wang , X. Xiao , L. Zhao , S. Wang , W.‐B. Liang , L. Zhang , J. Neurotrauma 2019, 36, 1018. 30261810 10.1089/neu.2018.5647

[42] S. Cao , M. Li , Y. Sun , P. Wu , W. Yang , H. Dai , Y. Guo , Y. Ye , Z. Wang , X. Xie , X. Chen , W. Liang , Nutrition 2022, 97, 111621. 35255397 10.1016/j.nut.2022.111621

[43] D. A. Long , K. Ghosh , A. N. Moore , C. E. Dixon , Brain Res. 1996, 717, 109. 8738260 10.1016/0006-8993(95)01500-0

[44] Q. Yang , M. Li , J. Liu , L. Zhang , R. Yuan , Y. Xu , J. Zheng , S. Cao , H. Dai , M. Liao , M. Lv , X. Chen , Y. Guo , X. Xie , L. Zhang , X. Chen , W. Liang , Nutrition 2023, 109, 111992. 36871445 10.1016/j.nut.2023.111992

