# Supplementary material for: Ferroptosis‐Mediated Hippocampal Neuronal Loss Post‐mTBI: Chromatin Accessibility Profiling and Single‐Nucleus Transcriptomics
Source: Adv Sci (Weinh). 2025 Dec 15;13(12):e12362. doi: 10.1002/advs.202512362 (PMC12948219; doi:10.1002/advs.202512362)
Supplement: Supplementary file 1 — Supporting Information [file ADVS-13-e12362-s005.docx]

Ferroptosis-Mediated Hippocampal Neuronal Loss Post-mTBI: Chromatin Accessibility Profiling and Single-Nucleus Transcriptomics

**Authors:** Manrui Li^1†^, Qiuyun Yang^2†^, Shengqiu Qu^1†^, Yang Chen^1^, Yang Shen^1^, Yang Xu^1,^ Xilong Lin^1^, Yihan Sun^1^, Ying Chen^1^, Meili Lv^3^, Lin Zhang^4^, Zengqiang Yuan^5^*, Weibo Liang^1^*, Xiameng Chen^2^*

**List of Supplementary Materials**

Supplementary Materials and Methods

Fig. S1 to Fig. S8

**Supplementary Materials and Methods**

**Read Alignment**

Raw base-call files (Illumina NovaSeq 6000) were demultiplexed with Cell Ranger to generate per-sample FASTQ files. For each library, gene-barcode matrices were produced with cellranger count against mm10 (GRCm38) using the 10× pre-mRNA reference and enabling intronic capture. Cell Ranger performs read alignment internally with STAR, corrects cell barcodes to the 10× whitelist, collapses UMIs, and generates filtered and raw feature-barcode matrices. Filtered matrices from each library were imported into R with Seurat::Read10× for downstream quality control, integration, and analysis

**snRNA-Seq Data Processing**

The Read10X function from the Seurat package was used to load the processed sequencing output files (barcodes.tsv, genes.tsv, and matrix.mtx) into R, followed by the creation of a Seurat object using the CreateSeuratObject function with the parameters "counts, min.cells = 3, min.features = 200". To quantify mitochondrial content in each nucleus, the PercentageFeatureSet function was used to calculate the proportion of genes with names starting with "mt-", which are of mitochondrial origin. Additionally, the percentage of hemoglobin-related transcripts was assessed to evaluate potential contamination from red blood cells, where genes beginning with "Hbb" and "Hba" were used to represent hemoglobin beta and hemoglobin alpha, respectively. Single-nucleus data were filtered using the following criteria to ensure high-quality data for downstream analysis: nFeature_RNA > 1,000, nFeature_RNA < 7,500, percent.mt < 10%, nCount_RNA < 18,000, nCount_RNA > 2,000, and percent.HB < 0.1%. These filtering thresholds ensured the retention of high-quality single-nucleus data for further analysis.

**Sequencing Data Integration and Dimensionality Reduction**

Data normalization and standardization were performed using the SCTransform function from the Seurat package, with the glmGamPoi method specified for modeling RNA reads data. The top 3,000 highly variable genes were selected as features for data integration. To ensure comparability across datasets, minimize batch effects, and preserve biological variability, the PrepSCTIntegration function was applied to standardize and scale the selected features. Once preprocessing was complete, principal component analysis (PCA) was conducted on each dataset using the RunPCA function in Seurat, leveraging the selected highly variable genes to reduce dimensionality and capture the major axes of variation. Dataset integration was performed using FindIntegrationAnchors and IntegrateData, both of which utilize the mutual nearest neighbors (MNN) algorithm to identify correspondences between datasets. This approach aligns similar cell populations across different samples while preserving unique biological signals within each dataset, facilitating effective data integration. The integration process used the top 20 PCs from PCA as anchors to maximize the retention of informative features. Following integration, the ScaleData function was applied to scale the integrated data, normalize gene expression levels, and remove unimportant sources of variation. PCA was then rerun on the integrated dataset, further reducing dimensionality while focusing on the top 20 PCs. Upon completing PCA, the FindNeighbors function was used for neighborhood identification, taking into account the retained PCs. Clustering was performed using the FindClusters function with a resolution parameter set to 0.1. After clustering, Uniform Manifold Approximation and Projection (UMAP) was applied using the RunUMAP function to visualize the data in a two-dimensional space.

**Cell-Type Annotation**

Cell-type annotation was performed using the FindAllMarkers function from the Seurat package to identify differentially expressed genes (DEGs) across clusters. Gene expression levels between clusters were compared using the Wilcoxon rank-sum test, with the following parameter settings: min.pct = 0.25, only.pos = TRUE, and logfc.threshold = 0.25. The resulting cluster marker genes were further filtered based on a p-value threshold of <0.05 for downstream annotation. Cell types were manually annotated based on reference markers from the CellMarker 2.0 database and commonly used cell markers reported in the literature. This approach allowed the identification and classification of seven major cell types, including Neurons, Oligodendrocytes, Astrocytes/Neural Stem Cells (Astrocyte_NSC), Microglia, Oligodendrocyte Precursor Cells (OPCs), Ependymal Cells, and Endothelial Cells. Finally, the cell-type annotation information was embedded into the main Seurat object under the "celltype" metadata.

**Differentially expressed genes (DEGs) Analysis**

Data normalization was performed using the NormalizeData function in Seurat, with LogNormalize specified as the normalization method. Following normalization, data were scaled using the ScaleData function. Differential expression analysis was conducted using the FindMarkers function on the normalized and scaled data. This function was applied separately to each major cell type identified in the study, comparing gene expression differences between the mTBI and sham conditions within each cell type.

**Neuronal Extraction, Dimensionality Reduction, and Cell-Type Annotation in snRNA-Seq Data**

Neurons were extracted from the main Seurat object using the subset function, isolating all Neuron cells. PCA was then applied to reduce dimensionality, and results were visualized using the DimPlot function. The top 15 PCs were selected for downstream analysis. Neuronal clustering was performed using FindNeighbors and FindClusters with resolution = 0.5, followed by further dimensionality reduction using UMAP. The results were visualized with DimPlot. Neuronal subtypes were annotated based on reference databases and commonly used hippocampal neuronal markers. Neurons were classified into the following subtypes: CA1, CA2, CA3, Dentate Gyrus Granule Cells (DG_GC), Interneurons (InN), Cajal-Retzius Cells (CR), Neuronal_subtype1, and Neuronal_subtype2.

**KEGG and GO Enrichment Analysis**

DEGs identified were used as input for enrichment analysis. KEGG pathway analysis was performed using the clusterProfiler package to identify significantly enriched metabolic and signaling pathways, with pathways having a p-value < 0.05 considered significant. For GO enrichment analysis, the clusterProfiler package was also utilized, categorizing results into three main functional groups: Biological Process (BP), Molecular Function (MF), and Cellular Component (CC). GO terms with a p-value < 0.05 were considered statistically significant. The results of KEGG and GO analyses were visualized using the ggplot2 package to facilitate interpretation and presentation of enriched pathways and functional categories.

**Construction and Scoring of Cell Death Pathway Gene Sets**

The apoptosis gene set was derived from genes included in the KEGG pathway. Gene sets associated with other forms of cell death were obtained from previously published literature and publicly available databases, covering the following cell death pathways: Ferroptosis (n=12), Cuproptosis (n=10), PANoptosis (n=27), Autophagy (n=15), Necroptosis (n=6), Entosis (n=34), Alkaliptosis (n=14), Lysosome-Dependent Cell Death (LDCD, n=5), Pyroptosis (n=21), Phagoptosis (n=7), Necrosis (n=15), Netosis (n=31), Parthanatos (n=19), and Oxeiptosis (n=21).

AUCell “active” calls were obtained with AUCell_exploreThresholds (method="global"); the knee-point threshold returned by the function was used per gene set. For group or subtype contrasts, per-cell AUCs were first aggregated as per-animal means within each neuronal subtype (pseudo-bulk by animal × subtype), then compared using Wilcoxon rank-sum (two groups) or Kruskal–Wallis with Dunn’s post-hoc tests (≥3 groups). P-values were Benjamini–Hochberg corrected across gene sets and subtypes, with FDR q<0.05 considered significant; we also report effect sizes (Cliff’s delta) alongside q-values.

**GSEA Analysis**

DEGs identified in the analysis were used as input for Gene Set Enrichment Analysis (GSEA). The clusterProfiler package was employed to evaluate the enrichment level of each gene set by calculating the association strength between gene sets and phenotypes. The Enrichment Score (ES) was computed to represent the rank distribution of each gene set within the gene expression list. Additionally, the Normalized Enrichment Score (NES) was calculated to account for differences in gene set size and provide a standardized measure of enrichment. Based on the enrichment results, enrichment plots were generated using the enrichplot package to visually represent the enrichment patterns of gene sets across the dataset.

**SCENIC Analysis**

SCENIC (version 1.3.1) was used to construct and analyze gene regulatory networks. First, the GENIE3 algorithm was applied to establish a co-expression network between transcription factors (TFs) and their target genes. GENIE3 infers potential gene regulatory relationships by analyzing gene expression data, providing a foundation for subsequent regulatory module analysis. Next, RcisTarget was used to identify TFs that directly bind to DNA. This analysis was performed using two databases: "mm9-tss-centered-10kb-7-species.mc9nr.feather" and "mm10-refseq-r80-500bp-up-and-100bp-down-tss.mc9nr.feather", which provide detailed information on TF binding sites, enabling accurate identification of potential direct regulatory targets for each TF. Subsequently, AUCell was used to quantify regulon activity scores in individual cells, allowing for the evaluation of regulon activity levels across different neuronal subtypes. After averaging and normalizing the regulon activity scores, the results were visualized using heatmaps and UMAP representations, providing a comprehensive view of regulatory network activity.

**ATAC-seq Data Processing**

Reads were aligned to mm10 (GRCm38) with bowtie2 (v2.x; --very-sensitive -X 2000); PCR duplicates and mitochondrial reads (chrM) were removed. Properly paired, uniquely mapped fragments (MAPQ ≥30) were retained. Peaks were called with MACS2 (v2.2.x) using paired-end mode (callpeak -f BAMPE -g mm -q 0.01). Reproducible peaks across biological replicates were defined by IDR (v2.0.4) with IDR < 0.05; the union of reproducible peaks formed the consensus peak set. Peak quantification used fragment counts per peak; differential accessibility was tested with DESeq2, and peaks with FDR (BH) < 0.05 were considered significant. Peak annotation employed ChIPseeker (TxDb.Mmusculus.UCSC.mm10.knownGene; promoter defined as -2 kb to +1 kb from TSS), and motif analysis used HOMER (findMotifsGenome.pl, mm10, -size 200 -mask) for known and de novo motif discovery. Aggregate TSS enrichment and peak-center profiles were generated with deepTools/ChIPseeker around TSS ±3 kb; browser tracks were created from CPM-normalized bigWig files.

**Frozen Tissue Sectioning**

Following cardiac perfusion with saline and 4% paraformaldehyde (PFA), mouse brains were collected and fixed overnight in 4°C PFA solution. The fixed brain tissue was then transferred to 30% sucrose solution and incubated until it fully sank. Subsequently, the tissue was embedded in OCT compound, rapidly frozen, and mounted on the sample holder of a cryostat (Leica CM1950). The tissue was sectioned into 10-μm thick coronal slices at -20°C. Sections were collected onto poly-L-lysine-coated glass slides and stored at 4°C for Nissl staining, Prussian blue staining, or immunofluorescence staining. Unused sections were stored at -80°C for future use.

**Paraffin Tissue Sectioning**

Human brain tissue was obtained from autopsy samples, and hippocampal specimens were immediately preserved in 4% PFA in PBS. Fixed tissues underwent dehydration using a graded ethanol series (70%, 80%, 95%, 100%), with each step lasting 1 hour. Dehydrated samples were then treated with xylene for clearing, followed by three rounds of paraffin infiltration, each lasting 2 hours. The infiltrated tissues were then embedded in paraffin blocks, cooled, and solidified for further processing.Paraffin-embedded brain tissues were sectioned into 5-μm thick continuous slices using a microtome (Leica RM2235). Sections were transferred to a water bath for flattening, then mounted onto glass slides and dried for storage. Before staining, sections underwent deparaffinization and rehydration. Deparaffinization was performed with xylene, followed by rehydration through a graded ethanol series (100%, 95%, 80%, 70%). After rehydration, sections were prepared for immunohistochemical (IHC) staining.

**Immunofluorescence Staining**

Prepared mouse brain frozen sections were removed from storage and allowed to equilibrate to room temperature for 30 minutes before staining. Sections were then washed three times with PBS (5 minutes each) to remove the protective medium and rehydrate the tissue. Permeabilization was performed by incubating the sections with 40 µL of 0.3% Triton X-100 at room temperature for 15 minutes, followed by three additional PBS washes (5 minutes each). To prevent nonspecific binding, sections were blocked in 3% BSA for 30 minutes before overnight incubation at 4°C with the following primary antibodies: Mouse anti-NeuN antibody (Cell Signaling Technology, Cat# 94403, 1:1000 dilution) and Rabbit anti-Tβ4 antibody (Thermo Fisher Scientific, Cat# PA5-100118, 1:100 dilution). The next day, sections were washed three times with PBS (5 minutes each) before being incubated in the dark at room temperature for 1 hour with Goat Anti-Rabbit IgG H&L (Alexa Fluor® 488, Abcam, Cat# ab150077) for Tβ4 detection and Goat Anti-Mouse IgG H&L (Alexa Fluor® 594, Abcam, Cat# ab150116) for NeuN detection. After secondary antibody incubation, sections were washed three times with PBS (5 minutes each), followed by nuclear staining with DAPI (10 μg/mL, Biosharp) for 10 minutes. Finally, sections were mounted using anti-fade mounting medium (Solarbio, Cat# S2100) and visualized using a Zeiss LSM 810 confocal microscope. Fluorescence signals from Alexa Fluor® 488 (green), Alexa Fluor® 594 (red), and DAPI (blue fluorescence) were captured using appropriate fluorescence filters, and multichannel image merging and post-processing were conducted with ZEISS ZEN lite image analysis software.

**Immunohistochemistry (IHC) Staining**

Human brain paraffin-embedded sections underwent standard deparaffinization and rehydration followed by antigen retrieval in citrate buffer (Solarbio, C1010, pH 6.0) using microwave heating for 20 minutes. After retrieval, sections were allowed to cool naturally at room temperature for at least 20 minutes, followed by three PBS washes (5 minutes each). To block endogenous peroxidase activity, sections were incubated in 3% hydrogen peroxide solution for 10 minutes. Non-specific background staining was minimized by blocking with 5% normal goat serum in PBS for 30 minutes. Sections were then incubated overnight at 4°C with the primary antibody: Rabbit anti-Tβ4 (Thermo Fisher Scientific, Cat# PA5-100118, 1:50 dilution). The staining procedure was performed using the Zhongshan Jinqiao Two-Step Detection Kit (Mouse/Rabbit Enhanced Polymer Method, Cat# PV-9000) according to the manufacturer’s instructions. Following DAB chromogenic detection, sections were washed with water, dehydrated through a graded ethanol series, cleared in xylene, and mounted with neutral resin. The stained sections were visualized and imaged using a BZ-X800 fluorescence microscope (KEYENCE, Tokyo, Japan).

**Western blot**

Hippocampi were homogenized in RIPA buffer with protease/phosphatase inhibitors, clarified by centrifugation, and quantified (BCA). Equal protein (200 µg) was separated by SDS-PAGE, transferred to PVDF, and probed with anti-Tmsb4x (Thermo Fisher Scientific, Cat# PA5-100118, 1:1000 dilution) and Gapdh (Bioswamp, Cat#MAB45855, 1:1000 dilution). Bands were visualized by ECL; densitometry (Tmsb4x/Gapdh) was performed in ImageJ; per-animal values were used for analysis.

**Chromatin immunoprecipitation and qPCR (ChIP-qPCR)**

Hippocampi from sham and mTBI (48 h) mice were minced on ice and cross-linked in 1% formaldehyde for 10 min; reactions were quenched with 125 mM glycine for 5 min. Nuclei were isolated and chromatin was sonicated to 200-500 bp. Equal amounts of chromatin were incubated overnight at 4 °C with anti-c-Jun antibody (proteintech, Cat#24909-1-AP, 1:50 dilution) or normal IgG (HUABIO, Cat# HA722127, 1:100 dilution) and Protein A/G magnetic beads (iGeneTech, Cat#C80662). Beads were washed sequentially with low-salt, high-salt, LiCl, and TE buffers, and complexes were eluted. Cross-links were reversed at 65 °C, followed by RNase A and Proteinase K treatment, and DNA purification. qPCR was performed with SYBR Green chemistry targeting a single amplicon (primer-F: GTTTGCTATAATTTCCGTGAGC, primer-R: ACTTAATTTTGAAGGGGCCGG) spanning an AP-1 motif in the Tmsb4x promoter. Signals were normalized as percent input (and expressed relative to IgG). Technical triplicates were averaged per animal.

**ELISA Analysis**

Hippocampal tissues were collected from male and female mTBI and sham mice (n=3 per group). Tissue homogenization was performed using a fully automated rapid sample grinder (Shanghai Jingxin, JXFSTPRP-24), followed by centrifugation to collect the supernatant for ELISA analysis. Protein concentrations for all samples were standardized using the Bradford protein assay.Tβ4 levels were measured using an ELISA kit (Quanzhou Ruixin Biotechnology Co., Ltd.), following the manufacturer's instructions. Absorbance (OD values) was measured using a microplate reader (Rayto RT-6100), and Tβ4 protein concentrations in hippocampal samples were calculated based on a standard curve. All measurements were performed in triplicate, including technical replicates for statistical analysis. For cell suspension samples obtained after FACS, cells were first pelleted by low-speed centrifugation (500 g, 4–5 minutes) to remove the supernatant. The cell pellet was then lysed using NP-40 Lysis Buffer (Solarbio, Beijing, China, J619-100) according to the manufacturer’s instructions. After centrifugation, the supernatant was collected for ELISA analysis, following the same protocol as for hippocampal tissue samples.

**Construction of c-Jun Overexpression Plasmid and Tmsb4x Promoter Overexpression Plasmid**

The c-Jun overexpression plasmid was constructed using the GV712 vector, which contains a CMV enhancer, multiple cloning site (MCS), SV40 promoter, and puromycin resistance gene. The target gene, mouse c-Jun (GenBank accession: NM_010591.2), was amplified by PCR using high-fidelity DNA polymerase with the primers Jun(70082-1)-p1 and Jun(70082-1)-p2 to ensure accurate amplification. The amplified c-Jun fragment was purified via gel electrophoresis and ligated into the GV712 vector, followed by transformation into E. coli via heat shock. Antibiotic selection was applied to obtain resistant clones, which were identified by PCR using primers Jun(70082-1)-p1 and Jun(70082-1)-p3, with an expected PCR product size of 1,158 bp. Positive clones were selected for plasmid extraction and sent for sequencing to confirm the correct insertion of the c-Jun sequence. Similarly, the Tmsb4x promoter overexpression plasmid was constructed using the GV238 vector, which contains an MCS and a firefly luciferase reporter gene. The target region, the mouse Tmsb4x promoter (GenBank accession: NM_021278.2-promoter), was amplified by PCR using the primers Tmsb4x(102995-1)-p1 and Tmsb4x(102995-1)-p2, with high-fidelity DNA polymerase to ensure accuracy. The amplified Tmsb4x promoter fragment was ligated into the GV238 vector, followed by heat shock transformation into E. coli. Resistant clones were selected using antibiotic-containing culture media and identified by PCR with primers KL102995-p3 and KL102995-p4, yielding an expected PCR product size of 1,014 bp. Plasmids from PCR-positive clones were extracted and sequenced to confirm the correct insertion of the Tmsb4x promoter into the vector. In all experiments, an empty vector control was included to eliminate potential background effects of the vector itself on experimental outcomes.

**Cultivation of HT22 Hippocampal Neuronal Cell Line**

The HT22 mouse hippocampal neuronal cell line (RRID: CVCL_0321, a gift from Dr. Xiao Xiao, sichuan university) was cultured in Dulbecco’s Modified Eagle Medium (DMEM) (Hyclone) supplemented with 10% fetal bovine serum (FBS, Hyclone) and 1% penicillin-streptomycin (Hyclone). Cells were maintained in a humidified incubator at 37°C with 5% CO₂.

**Cell Transfection**

For transfection, siRNA or plasmid DNA was mixed with Lipofectamine 3000 transfection reagent (Thermo Fisher) according to the manufacturer’s protocol. siRNA or plasmid DNA was pre-mixed with Lipofectamine 3000 (and P3000 enhancer, if applicable) and incubated at room temperature for 20 minutes to form siRNA/DNA-lipid complexes. The transfection complexes were then added to the cells and gently agitated to enhance mixing and uptake. After transfection, cells were incubated at 37°C for 48 hours to allow for siRNA/plasmid expression.

**Luciferase Assay**

Luciferase activity was measured using the Luciferase Reporter Gene Assay Kit (Yeason, Cat# 11401ES60). Transfected cells were collected, washed with 1× luciferase assay buffer, and lysed according to the kit instructions. The cell lysates were then transferred to a white 96-well microplate, and luciferase substrate was added. Relative luminescence units (RLU) were measured using a TECAN Spark microplate reader. Each experimental group included three technical replicates to ensure data reliability and reproducibility.

**Trypan Blue Staining**

Trypan blue staining was performed using Trypan Blue Staining Solution (Beyotime, Cat# ST798) according to the manufacturer's instructions. After cell collection, cells were gently washed once with PBS and detached using trypsin digestion. The cell suspension was then mixed with an equal volume of Trypan blue solution and incubated for 3–5 minutes. The stained cell suspension was loaded into the Countstar BioTech automated cell counter, which automatically distinguishes live and dead cells using image analysis technology, recording the total cell count and percentage of viable cells for each sample.

**CCK-8 Assay**

Cell viability was assessed using the Cell Counting Kit-8 (CCK-8, Dojindo, Cat# CK04) following the manufacturer's protocol. After experimental treatment, 10 µL of CCK-8 solution was added to each well, and the plate was incubated at 37°C for 1 hour. Absorbance was then measured at 450 nm using a microplate reader, and the mean OD value for each treatment group was calculated after subtracting the mean OD value of the blank control. Each experimental group was analyzed in triplicate to ensure data reliability.

**Mitochondrial Morphology Analysis Using MitoTracker Staining**

To assess mitochondrial morphology, HT22 cells were seeded onto a 24-well glass-bottom culture plate suitable for microscopic imaging. After experimental treatment, cells were stained with MitoTracker Red CMXRos (Invitrogen, Cat# M7512). A 1 mM stock solution was prepared according to the manufacturer’s instructions and diluted 1:5000 in DMEM to create the working solution. Cells were incubated in the MitoTracker working solution at 37°C for 30 minutes. After staining, cells were gently washed 2–3 times with pre-warmed (37°C) PBS, followed by the addition of DMEM complete medium containing 10% FBS to cover the cells before imaging. Mitochondrial morphology was visualized and imaged using a Zeiss LSM 810 confocal microscope in Airyscan mode (Ex/Em: 579/599 nm).

**Fluorescence-Activated Cell Sorting (FACS)**

Hippocampal tissue was isolated from transgenic mice expressing tdTomato fluorescent protein in neurons. Following cardiac perfusion with saline, the brain was rapidly extracted, and the hippocampus was dissected. The hippocampal tissue was washed with ice-cold Hanks' Balanced Salt Solution (HBSS) to remove debris. The cleaned tissue was then transferred into a trypsin-containing solution, finely minced, and incubated at 37°C with gentle shaking for 15 minutes to dissociate single cells. To terminate digestion, DMEM supplemented with 10% FBS was immediately added. The cell suspension was then filtered through a 40-µm cell strainer and centrifuged at 300 g for 5 minutes to collect cells. The pelleted cells were resuspended in PBS containing 1% BSA and subjected to FACS using a BD Melody cell sorter. The cell suspension was loaded into the sample chamber, and tdTomato fluorescence was detected using the PE (Phycoerythrin) channel. Before sorting, forward scatter (FSC) and side scatter (SSC) thresholds were optimized in the flow cytometry software to distinguish live cells from dead cells and debris. A fluorescence threshold was set in the PE channel to differentiate tdTomato-expressing cells from non-expressing cells. Live tdTomato-positive cells were automatically sorted and collected into a collection tube containing culture medium for further analysis.

**GSH Assay**

Hippocampal tissue or cell samples were homogenized in pre-chilled PBS and subjected to high-speed centrifugation (10,000 g, 4°C, 10 minutes) to remove cell debris. The resulting supernatant was collected for analysis. The GSH and GSSG Detection Kit (Beyotime, Cat# S0053) was used according to the manufacturer's protocol. After preparing the detection working solution, samples were incubated at room temperature for 25 minutes, with absorbance measured every 5 minutes at 412 nm using a microplate reader. The total GSH and GSSG concentrations in the samples were quantified based on a standard curve, and GSH levels were normalized to total protein concentration determined by the BCA protein assay, expressed as µmol/mg protein.

**MDA Assay**

Hippocampal tissue or cell samples were homogenized in pre-chilled PBS and centrifuged at 10,000 g, 4°C, for 10 minutes to remove cell debris. The MDA Detection Kit (Beyotime, Cat# S0131M) was used according to the manufacturer's instructions. The sample lysates were mixed with the TBA reagent and antioxidant solution provided in the kit at the recommended ratio, followed by incubation in a metal bath for 15 minutes. After heating, the samples were allowed to cool to room temperature, then centrifuged at 10,000 g for 10 minutes to remove unreacted macromolecules and precipitates. Absorbance was measured at 532 nm using a microplate reader, and MDA levels were quantified based on a standard curve generated from known MDA concentrations. MDA results were normalized to total protein content, expressed as µmol/mg protein.

**Nissl Staining**

Paraffin-embedded hippocampal tissue sections were subjected to standard deparaffinization and rehydration. Nissl staining was performed using the Nissl Staining Solution (Beyotime, Cat# C0117). Rehydrated sections were incubated in Nissl staining solution for 10 minutes and then gently rinsed with distilled water to remove excess stain. The sections were dehydrated, cleared in xylene, and mounted with neutral resin. Stained sections were visualized and imaged using The EVOS FL Auto Imaging System.

**Perl’s Staining**

Paraffin-embedded hippocampal tissue sections were subjected to standard deparaffinization and rehydration. Iron ion staining was performed using the Enhanced Prussian Blue Staining Kit (Solarbio, Cat# G1428). Perls’ working solution was prepared according to the manufacturer’s instructions, and sections were incubated at 37°C for 20 minutes. After three washes with PBS, DAB-enhanced working solution was applied, followed by an additional 37°C incubation for 20 minutes. Sections were then washed in distilled water for 10 minutes, dehydrated through a graded ethanol series, cleared in xylene, and mounted with neutral resin. Stained sections were visualized and imaged using The EVOS FL Auto Imaging System.

**Transmission Electron Microscopy (TEM)**

Mice were anesthetized and perfused with 0.1 mol/L sodium cacodylate containing 4% paraformaldehyde (PFA) and 3% glutaraldehyde (GA); hippocampi were dissected and post-fixed overnight at 4 °C in 2% PFA/2.5% GA. Cultured neurons were briefly trypsinized (<1 min), pelleted (1,000 rpm, 2 min), pre-fixed in a 1:1 mixture of 3% GA and 0.1 mol/L PBS for 5 min at 4 °C, then transferred to 3% GA as the primary fixative. Cell pellets and hippocampal tissue were processed together: dehydrated through an acetone gradient, embedded in epoxy resin, and sectioned at 70-90 nm on a Leica EM UC7 ultramicrotome (Leica, Teaneck, NJ, USA). Ultrathin sections were contrasted with uranyl acetate and lead citrate, and imaged on a Hitachi HT7800 TEM (Hitachi, Tokyo, Japan). Images were acquired with the integrated digital camera under identical acquisition settings across groups.

**MRI**

MRI scanning was performed 48 hours post-injury using a 7 Tesla Bruker Biospec USR 70/30 MRI system (Bruker Biospin GmbH, Ettlingen, Germany). To assess tissue water content, T2-weighted RARE sequences were used with the following parameters: repetition time (TR) = 5209.436 ms, echo time (TE) = 60 ms, and field of view (FOV) = 20 × 20 mm. The acquisition matrix was set to 256 × 128, covering 25 slices, each with a thickness of 0.5 mm, using 8 averages and a RARE factor of 8. Relative hippocampal water content was calculated as hippocampal T2 signal intensity / whole-brain T2 signal intensity. To evaluate hippocampal metal ion deposition, T2-weighted imaging (T2*WI) was performed using a gradient echo sequence, a susceptibility-weighted technique capable of detecting paramagnetic substances such as deoxyhemoglobin, ferritin, and hemosiderin. The scanning parameters were as follows: FOV = 20 × 20 mm, number of slices = 10, slice thickness (THK) = 0.5 mm, TR = 150 ms, TE = 6 ms, averages = 4, and repetitions = 1. The total scan duration was 1 minute and 43 seconds. Hippocampal metal ion deposition density was calculated as hippocampal T2* signal intensity / whole-brain T2* signal intensity.

**Hippocampal Microinjection**

Following anesthesia, mice were placed in a stereotaxic frame, with body temperature maintained using a heating pad. The hippocampal injection site was determined based on the mouse brain atlas. Microinjections were performed using a KDL sterile disposable syringe (1.0 mL, U-100, Type 7), pre-flushed with PBS to remove air bubbles. Tβ4 protein (50 µg, MCE, Cat# HY-P72776) was dissolved in 100 µL sterile normal saline (NS) and aliquoted for storage. One hour after controlled cortical impact (CCI), 1 µL of Tβ4 protein solution was injected bilaterally into the hippocampus at the following stereotaxic coordinates (relative to Bregma): AP = -2.3 mm, ML = ±1.3 mm, DV = -2.0 mm. The injection rate was set at 0.2 µL/min, with a total injection duration of 5 minutes per site. After injection, the needle was left in place for 10 minutes before being withdrawn. The scalp was then sutured to complete the procedure.

**Supplementary Figures**


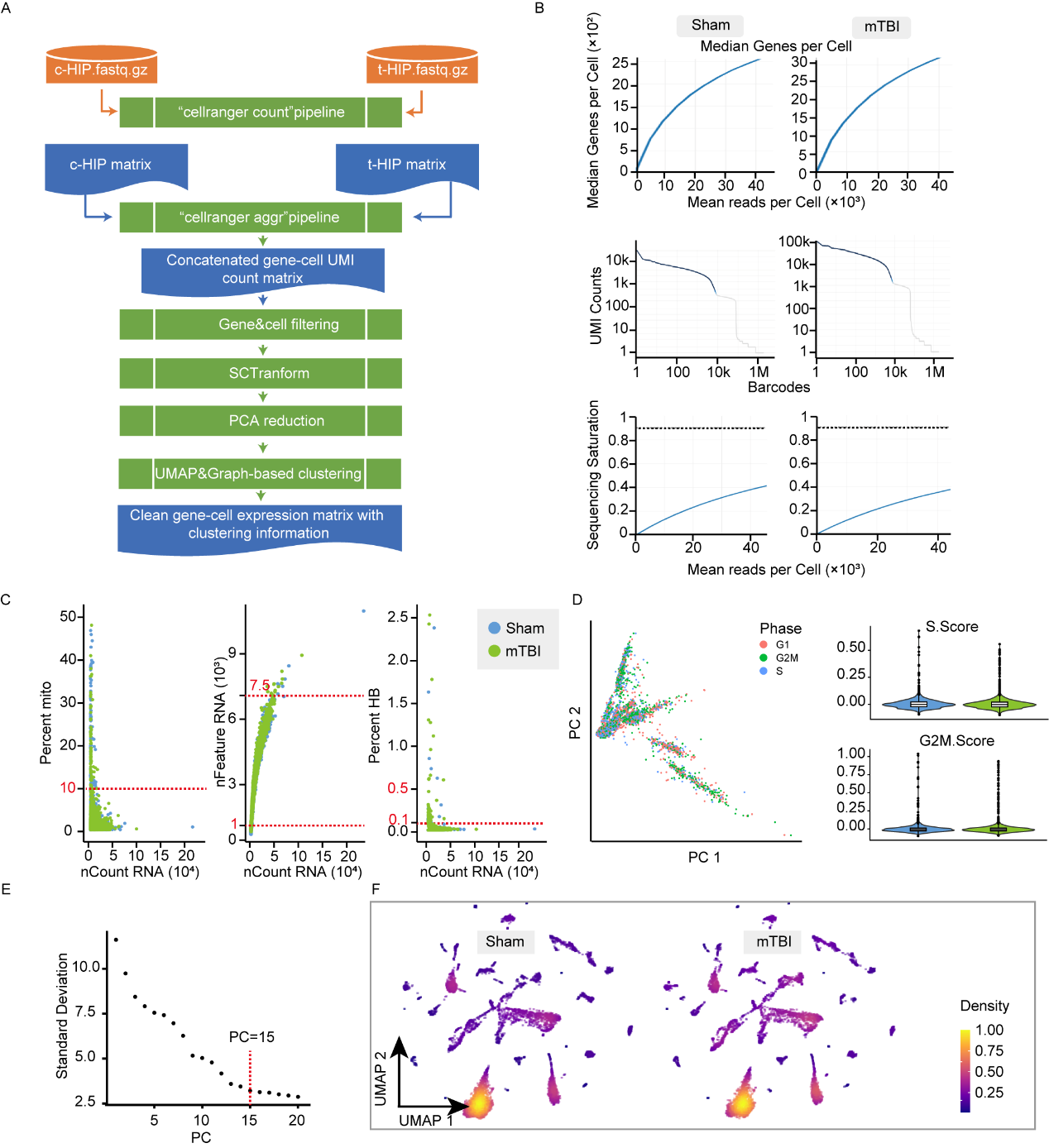


**Fig. S1: Single-Nucleus Sequencing Data Processing Workflow and Quality Control Criteria**

(A) Overview of the raw data output from single-nucleus RNA sequencing and the brief processing workflow. (B) Summary of data processing results using Cell Ranger. (C) Filtering criteria for mitochondrial gene proportion (Percent.mito), number of detected genes (nFeature RNA), and red blood cell gene proportion (Percent HB), with red dashed lines indicating the filtering thresholds. (D) Cell cycle distribution across different phases (G1, G2M, and S) and a violin plot showing S-phase and G2M-phase scores. (E) Standard deviation distribution of principal components (PCs), with the top 15 PCs selected for further analysis. (F) UMAP plot and density distribution of Sham and mTBI groups.


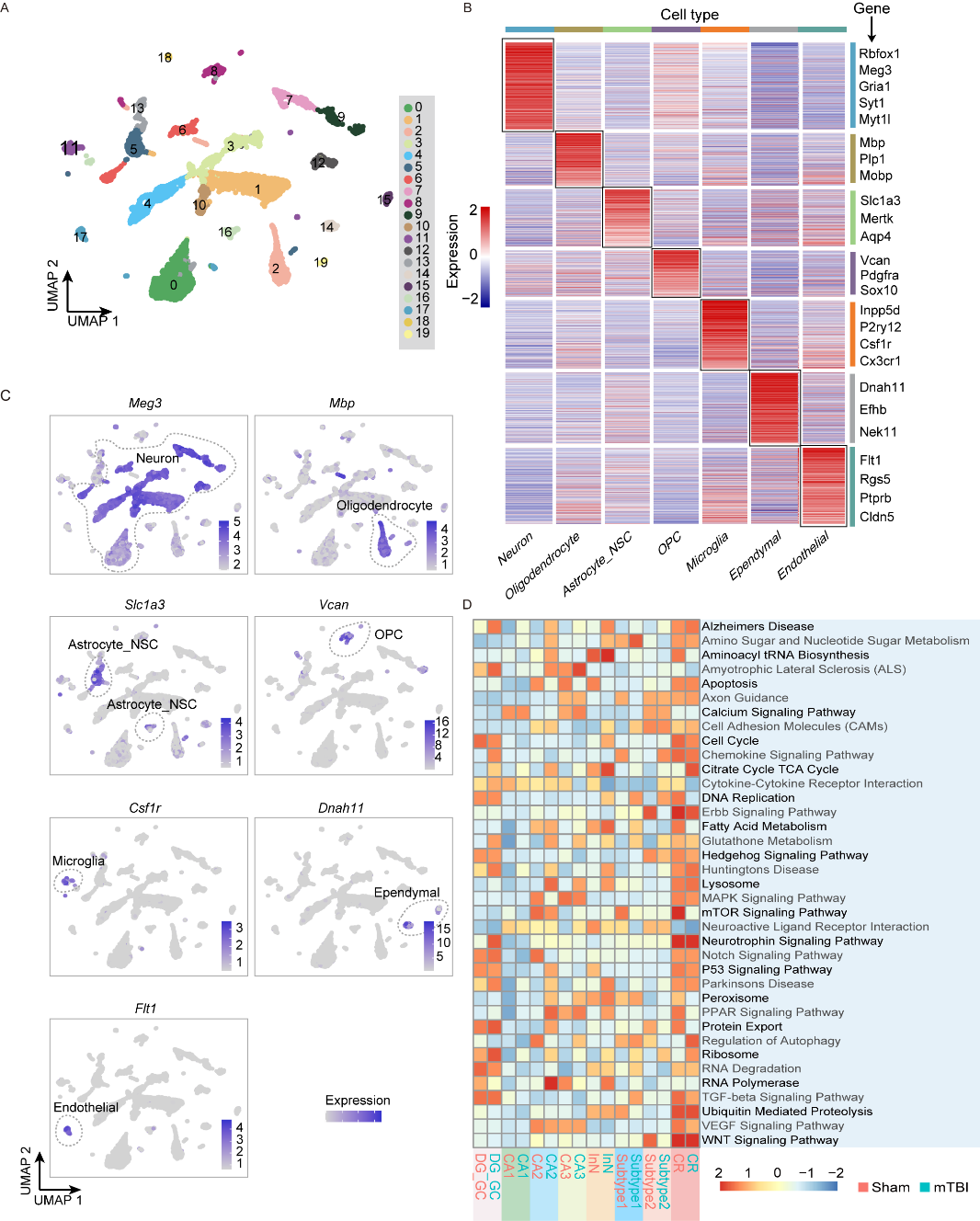


**Fig. S2: Identification of Cell Clusters and Functional Analysis of Neurons**

(A) UMAP plot displaying the distribution of all cells, grouped into 20 clusters. (B) Heatmap showing gene expression profiles across different cell types. (C) UMAP plot illustrating the distribution of cell type-specific markers. (D) Pathway activation analysis of different neuronal subtypes in the mTBI and Sham groups.


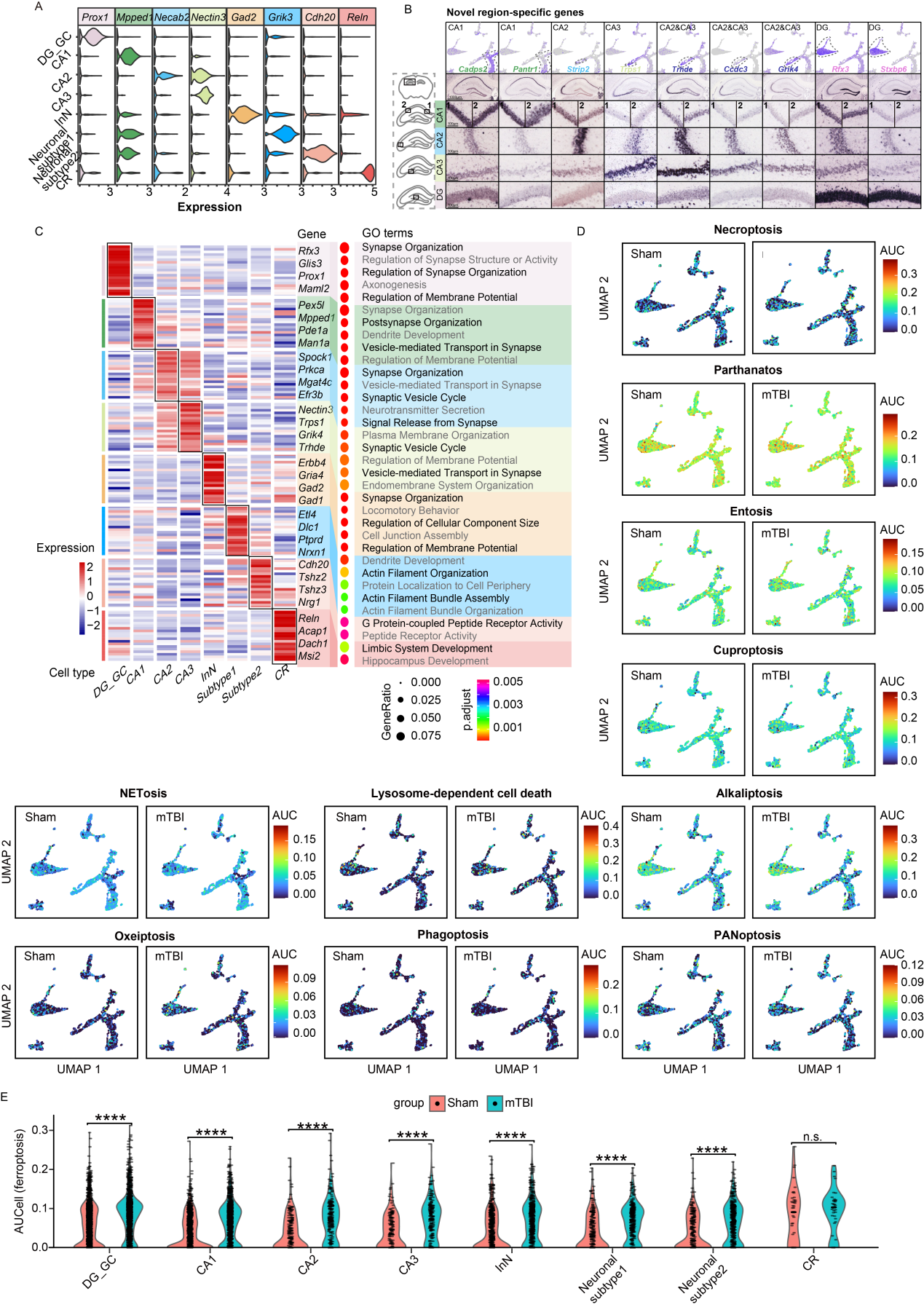


**Fig. S3: Functional Differences and Cell Death Pathway Activation in Neuronal Subtypes After mTBI**

(A) Violin plots showing the expression of subtype-specific markers in different hippocampal neuronal subtypes. (B) Localization of newly identified marker genes from our neuronal subtype analysis within the Allen Brain IHC database. (C) Heatmap displaying gene expression profiles across different neuronal subtypes. (D) Activation of various cell death pathways in neurons from mTBI and Sham mice, with AUC pathway activation scores mapped onto the UMAP plot. (E) AUCell ferroptosis module scores by neuronal subtype (animal-level summaries).
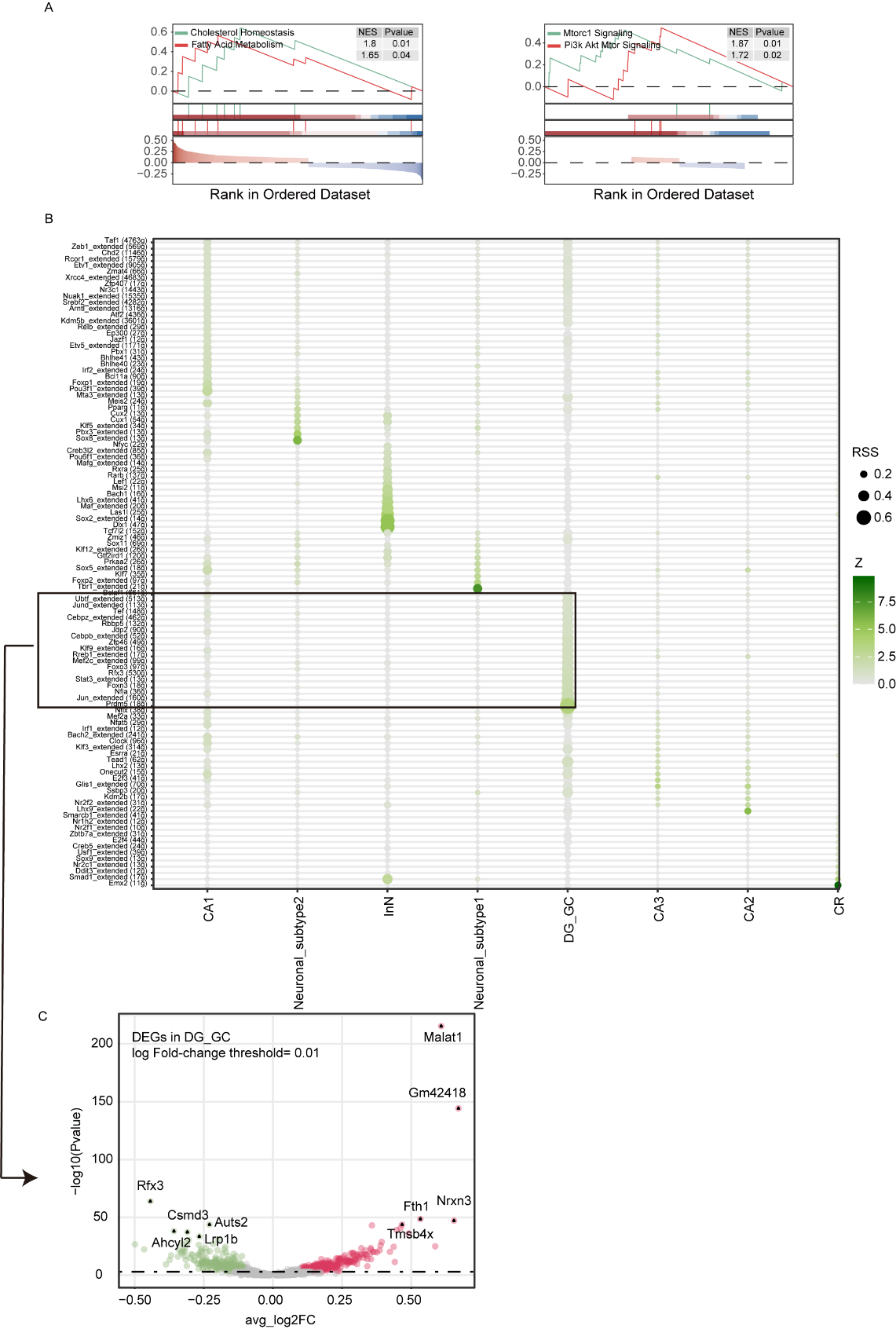


**Fig. S4: GSEA, SCENIC, and Differential Gene Expression (DEG) Analysis Results**

1. Selected GSEA pathways that are upregulated in the mTBI group. (B) SCENIC analysis showing changes in transcription factor activity following mTBI, where circle size represents RSS scores, and shades of green indicate significance levels (Z-values). (C) Volcano plot depicting differentially expressed genes (DEGs) in DG_GC cells.
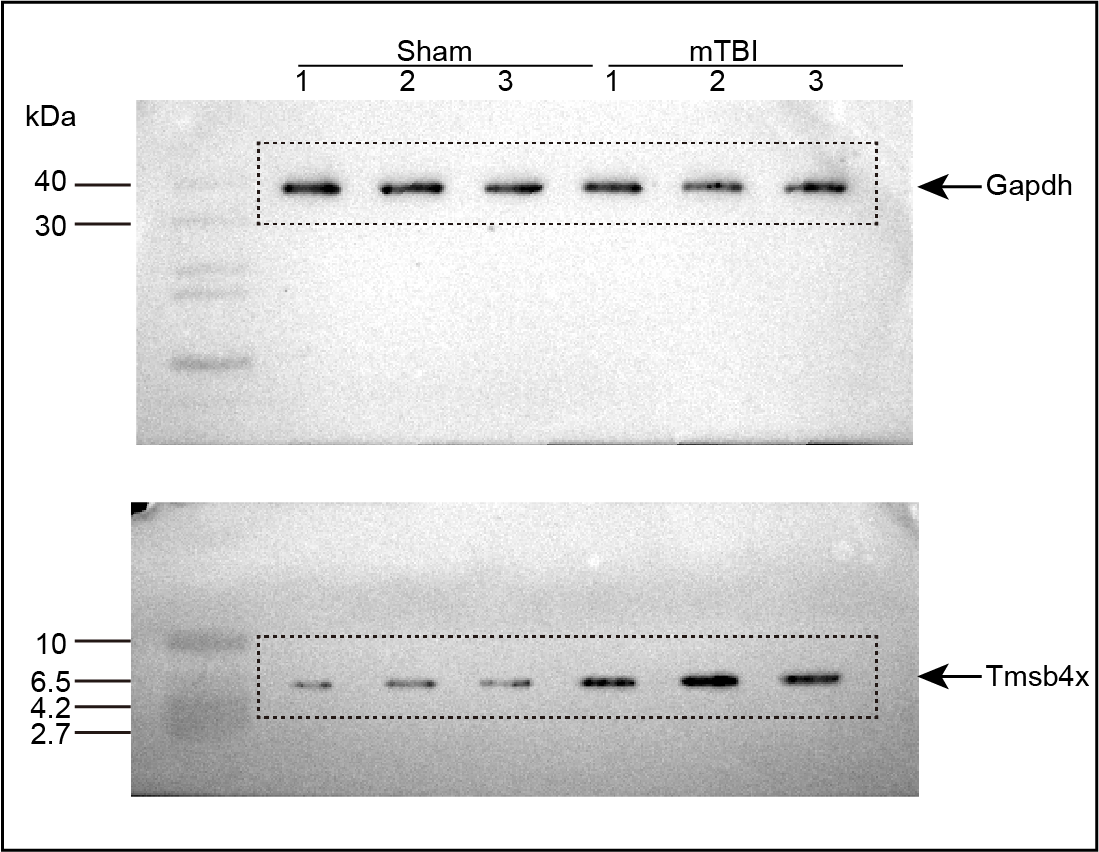


**Fig. S5: Original western blot images of this manuscript.**


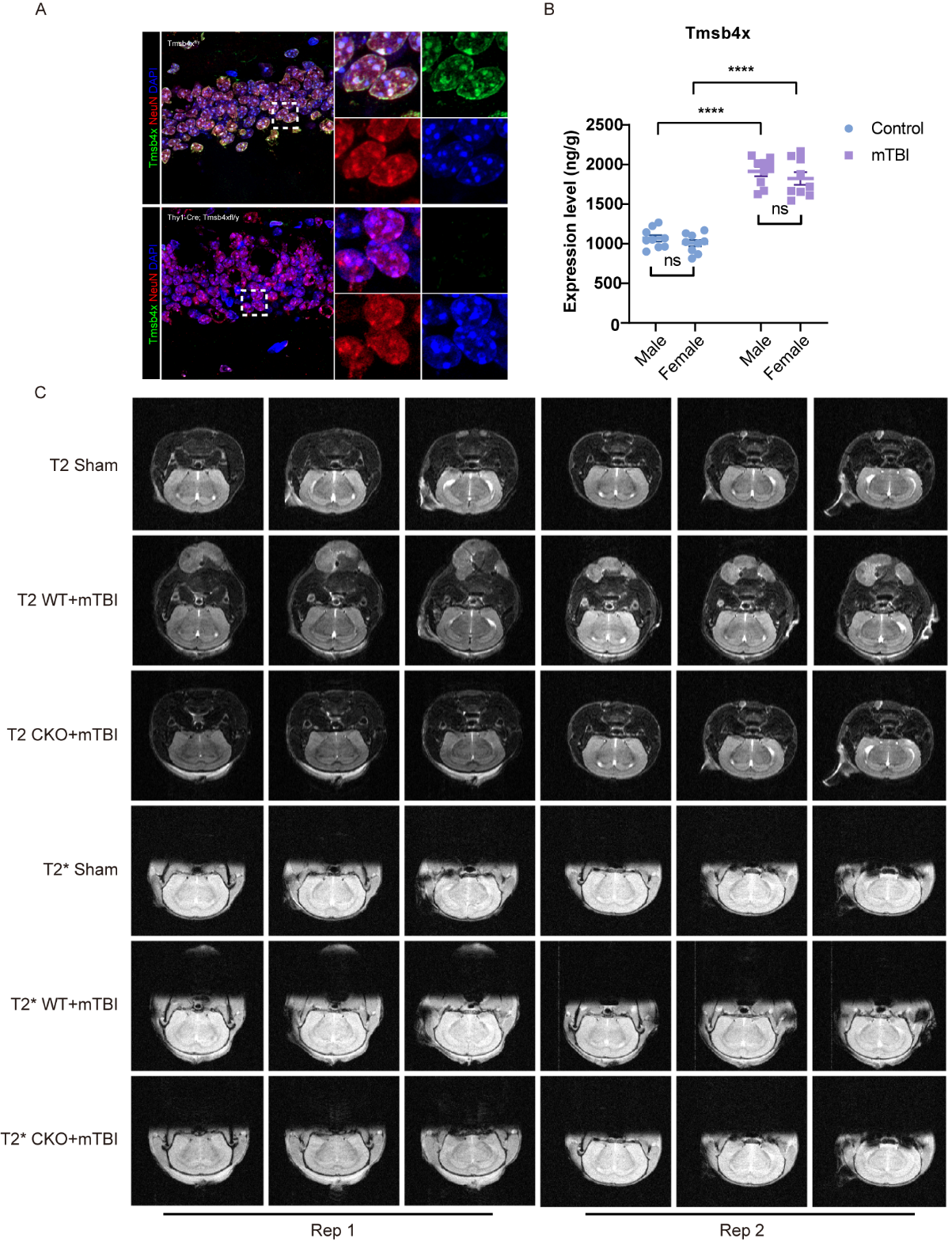


**Fig. S6: Validation of Tmsb4x Knockout in CKO Mice and Complete MRI Imaging**

(A) Immunofluorescence staining of hippocampal sections from Tmsb4x-CKO and control mice. (B) ELISA analysis of Tmsb4x expression in the hippocampus of male and female mTBI and control mice. (C) T2 and T2 MRI signal acquisition results* for CKO + mTBI, WT + mTBI, and Sham mice.


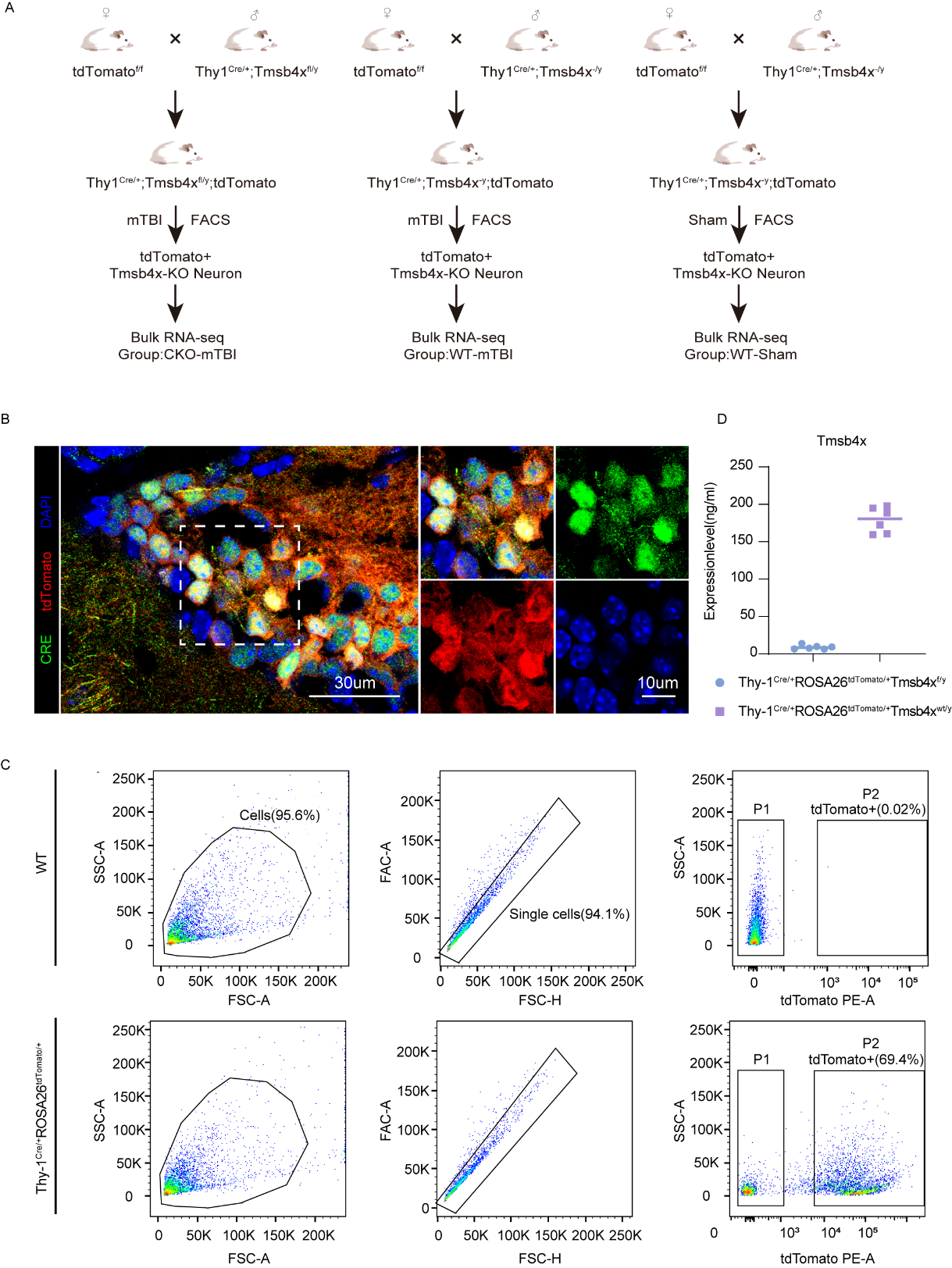


**Fig. S7: Mating Strategy, Grouping, and Validation of Thy-1Cre/+ROSA26tdTomato/+ Tmsb4x f/y Mice**

(A) Breeding strategy and experimental grouping of Thy-1^Cre/+^ROSA26^tdTomato/+^ Tmsb4x ^f/y^ mice. (B) Immunofluorescence staining confirming co-localization of CRE and tdTomato expression. (C) FACS analysis showing the sorting results of tdTomato-positive neurons.


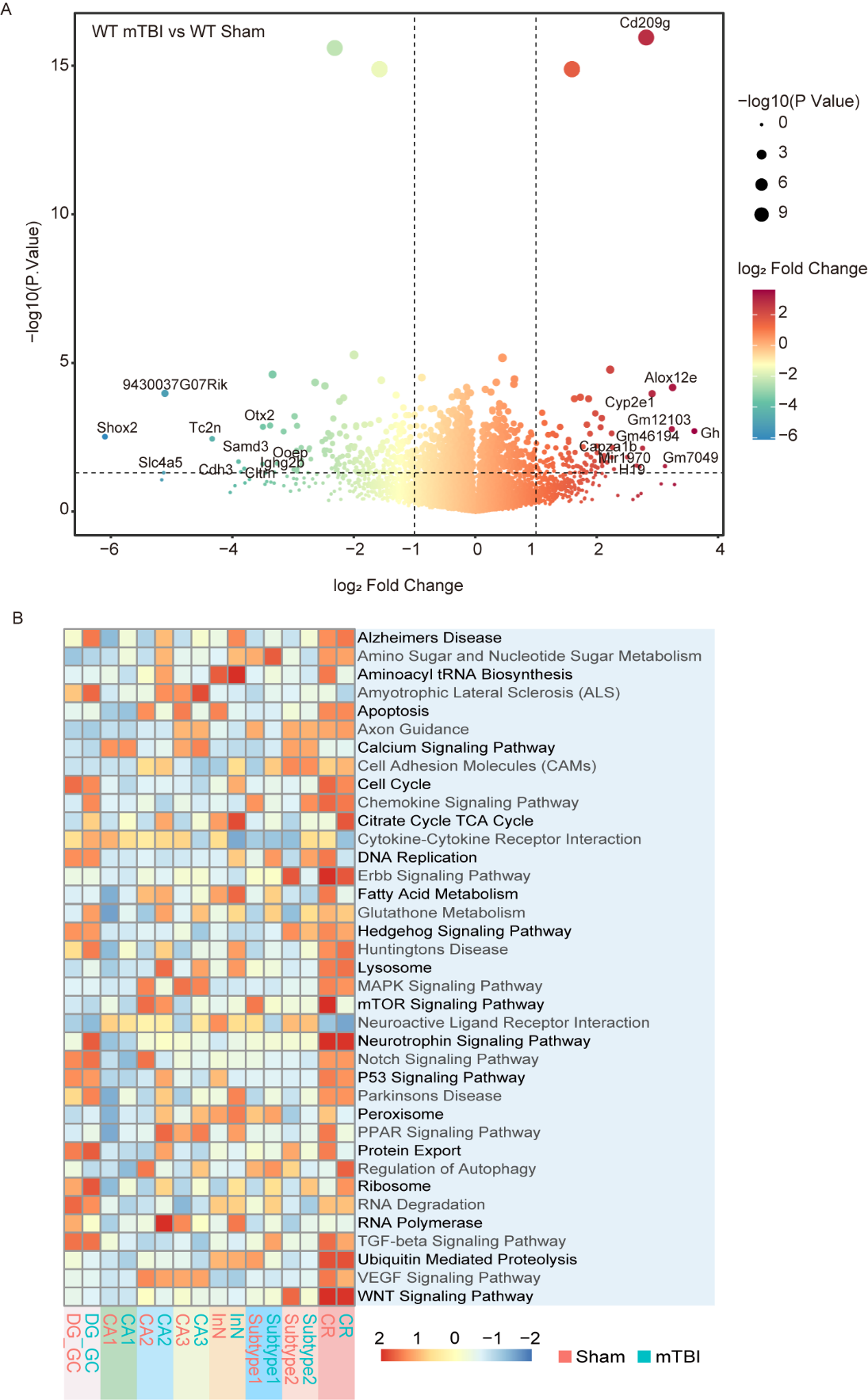


**Fig. S8: Bulk RNA Sequencing Data Analysis of Neurons in Tmsb4x-CKO Mice**

(A) Volcano plot of differentially expressed genes (DEGs) from bulk RNA sequencing of neurons in mTBI and Sham mice. (B) KEGG pathway enrichment analysis of DEGs in neurons from mTBI and Sham mice.

**Fig. S9: MitoTracker imaging for the co-silencing experiments.**

**
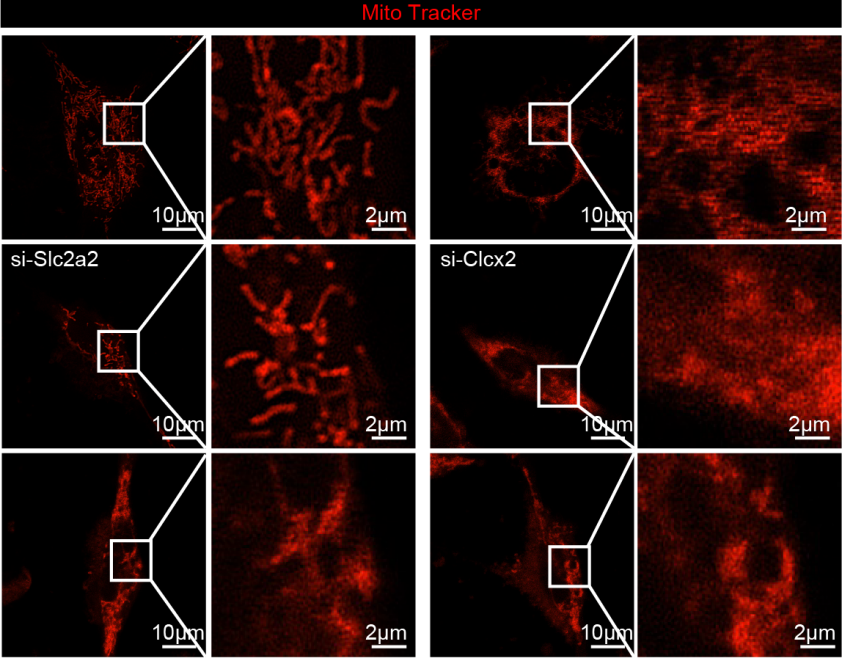
**

Representative MitoTracker images acquired under identical exposure/gain across groups (Blank, si-Tmsb4x, si-Slc2a2+si-Tmsb4x, si-Cxcl2+si-Tmsb4x, si-Card11+si-Tmsb4x, and si-Tnfsf13b+ si-Tmsb4x). Scale bars as indicated.
