# Supplementary material for: Ferroptosis‐Mediated Hippocampal Neuronal Loss Post‐mTBI: Chromatin Accessibility Profiling and Single‐Nucleus Transcriptomics
Source: Adv Sci (Weinh). 2025 Dec 15;13(12):e12362. doi: 10.1002/advs.202512362 (PMC12948219; doi:10.1002/advs.202512362)
Supplement: Supplementary file 3 — Supplemental Table Legends [file ADVS-13-e12362-s007.docx]

**Table legends**

**Table S1:List of marker genes for cell cluster identification in the hippocampus**

**Table S2: List of marker genes for cell type classification in the hippocampus.**

**Table S3: List of marker genes for hippocampal neuronal subtypes.**

**Table S4: Statistical data on the proportions of each neuronal subtype in the mTBI group and sham group.**

**Table S5: List of gene sets for 14 cell death pathways.**

**Table S6: Summary of activation scores of cell death pathways in different neuronal subtypes .**

**Table S7: Mitochondrial damage-related genes regulated by transcription factors c-Jun and Rfx3 and their chromatin accessibility changes in ATAC-seq analysis.**

**Table S8: List of primer sequences used in qPCR experiments.**
