# Supplementary material for: Ferroptosis‐Mediated Hippocampal Neuronal Loss Post‐mTBI: Chromatin Accessibility Profiling and Single‐Nucleus Transcriptomics
Source: Adv Sci (Weinh). 2025 Dec 15;13(12):e12362. doi: 10.1002/advs.202512362 (PMC12948219; doi:10.1002/advs.202512362)
Supplement: Supplementary file 2 — Supplemental Data File [file ADVS-13-e12362-s009.zip › Data file S1.DOC]

**Construction of overexpression vectors**

**gene information**

Gene：Tmsb4x(NM_021278.2-promoter)

Species：Mouse

**Vehicle information：**

Vehicle：GV238

Component sequence：MCS-firefly_Luciferase

Cloning site：KpnI / XhoI

Control number：CON080

Vehicle mapping：http://www.genechem.com.cn/service/index.php?ac=gene&at=vector_search&keyword=GV23

1. **Acquisition of target gene fragments**
2. **Primers**

| **ID** | **seq** |
| --- | --- |
| Tmsb4x(102995-1)-p1 | TTTCTCTATCGATAGGTACCGAACACATTTATTCTTTTCTTC |
| Tmsb4x(102995-1)-p2 | CTTAGATCGCAGATCTCGAGTTATGTGTGCTTCTATGCAAATG |

Primer description: Containing exchange paired bases, enzyme cleavage site, and part of the 5' end sequence of the target gene for PCR fishing for the target gene.

2.1 **PCR results**

Size of PCR products： 2070

**3. Recombinant plasmid construction**

3.1 **Exchange of products into linearised expression vectors**
3.2 **Primers for PCR identification**

| **ID** | **seq** |
| --- | --- |
| KL102995-p3 | TCTACCTGAATCGTCCACAT |
| KL102995-p4 | ACCAGGGCGTATCTCTTC |

3.3 **PCR identification of recombinant clones**

Positive transformant PCR product size：1014

**4.Sequencing results of positive clones and analysis of results：**

**Comparison results：**

CCCAGTGCAAGTGCAGGTGCCAGAACATTTCTCTATCGATAGGTACCGAACACATTTATTCTTTTCTTCTCAACTTTCTATTTTAAATAAGAAGAGAACACAGTGCTCGCCATTGTCCCAAGAACTTGATCTTCCCACAACTCCTACAGTTCCTTATCACCACTTGTCCACTTTACAGAGAAGAGTTAAGAGAAGAATGATCGACAGGAGGACCCGGACACTTTTAAGACAAACCCTCTTATTTGCATTTCATTTTATCCGGATCACCCTTTCTTTTCTATTTTTCTGCCCAGTGGGCAAGGGAAGCCCGTTTACAGAGGACTCAGTTCCCAGGGCAAGTTGCTTCCTCTCTTACAGGCTGCCTGCTCTTTTTCAGTCAGCAGCTAAAATAATCTGCCTGCTACGCTGTTACGAAAGTAGAAAATTCTAGATGTTTACAGATTCCACTCTTCAGTCTCCTCTGGCCGCATAACTTCTGTAAGGGCTAATCTTTCCTAAGGCAAACAGGCAACCAGGGTCAAGGCGATAGCATTCCTTTGTCATAGGAAGACACGTACACGGTGACAGCTGAGTGAAGGAAGGGACACTGGAATCCAATGACCCACCGAAATGGTGTCAGTGTAACTGCAGCTGCCAAGCAGAAGTCAGAAATGCACCCTGCAAGATGTGTTCTGTGTTTCAACAATGCAGTGAACTCCCTCCAAGTGGACATGGGGAGAGAGAGGATGTGTTACCAGAGAAGTGACATCTAATGCGCGCTCAAACAGGTCTAGGGCCCCATCCAAAGGGCAGAAAGGCAGTCTCCCAAGTAGAGCATTAACTGGGGAACCACATGGGTCACCTGCTCCTCCCTACCCCCATCTCCTTCCTCCCAAGTAAAAGGGAAGTAGATGAAGAAATGGGGAAGTCCAAGCTGCTCCAGCAGCGGCAGTTTAGGGTCCCTAATAACACTTGACATTACCAGAGAGGCAAAGCCTCTGCCATAAAAATGCTTAGCAAAGGCTTTCTGGAAACATCCCTCCAGGGCACAGAAAAGGAGAAGGAATGTTTTATAGTTCTCTGTGTCTCAGATATAATCCCAGCGATGCCCTTTTAAAAATATCCTACGGAGCTACCTCCAACCACCTGCCACTTGCAGGCACAAGCTTGGTATCTGTAATTACTTTTTAGTAGCAAGTGGGTCCCTGCGACTTACGGACTCAGGTGCCATTTTGAAACCGGCACATCTCTACCTGAATCGTCCACATGTGTTGCAGGGAAAACCTTCCCTTCAAAAGCTAGTTCGCGCTTCAGCTTCAATCCTCAGATAATAGAGCTGATGGACTTGTGGTCTTTGGGAATCCTTGGCTCCCATTAGCAATCACTTTTAATTGATGAAAATAAGAAAAGGAAAATGAACTTCCTTATTGTCAGTCAGTAACAGAAGTTGACTTTGACTTAATTTTGAAGGGGCCGGCTGTGTTCTCTCTGAAGCCCCCTTACACCTAGCAAATGTTAGGCACTTGACTCGATTGCTCACGGAAATTATAGCAAACAATACAAATGCTGCGGCGCTAAAACTTTTCTTTTAAAAATCCACTCAAGGATCCTTTATCGCCCTCTCAAATCAAGGGGGAGACAAGCGAGGGAGACAAAGAGGGCCGGGTGGGGCTGTCCACTGGTCTGAAAGCTGAGTCATTGGAATCAGACCCCAGCAGCTCTTGTATGAGGGACACCTCACCCTATCTTGGGGGCGTTGGCTGCAGACAAAGCCGGTTTCAAAGTAAAGAAAAGCCTGTCCCCTTAGGTGCCACTCAGGCCATTAGTTATCACGCATTGAACCTTAAGAACAGGTTTGGGCGGCTGCTGCAAAAGTTCATGATAATAAAATAGACGTCAAATGGGGGGGAAAGCATTTTGAAGGCGTTTGTTCCTTTTCTAGTTCCGCCATCGATGTGGTTCAAAGGCGCTGGTGAATGGGGATGTGGGGGTTGGGAGGGGGTCTGACAGCCTCCGAACGCCCTTCCGGAGATGCGGCGCGAAGGAGTTAAGCCGGACTACGAGTGACATCACCTCATTTGCATAGAAGCACACATAACTCGAGATCTGCGATCTAAGTAAGCTTGGCATTCCGGTACTGTTGGTAAAGCCACCATGGAAGACGCC

**Comparison result description: the sequencing reads through without any errors**
