# Supplementary material for: Ferroptosis‐Mediated Hippocampal Neuronal Loss Post‐mTBI: Chromatin Accessibility Profiling and Single‐Nucleus Transcriptomics
Source: Adv Sci (Weinh). 2025 Dec 15;13(12):e12362. doi: 10.1002/advs.202512362 (PMC12948219; doi:10.1002/advs.202512362)
Supplement: Supplementary file 2 — Supplemental Data File [file ADVS-13-e12362-s009.zip › Data file S2.DOC]

**Construction of overexpression vectors**

**gene information**

Gene：Jun(NM_010591.2)

species：Mouse

**Vehicle information：**

Vehicle：GV712

component sequence：CMV enhancer-MCS-SV40-puromycin

cloning site：XbaI / XbaI

Control Number：CON520

Vehicle mapping：http://www.genechem.com.cn/service/index.php?ac=gene&at=vector_search&keyword=GV71

1. **Acquisition of target gene fragments**

**2. Primers**

| **ID** | **seq** |
| --- | --- |
| Jun(70082-1)-p1 | GTTTAAACGGGCCCTCTAGACGCCACCATGACTGCAAAGATGGAAAC |
| Jun(70082-1)-p2 | CAGCGGTTTAACTATCTAGATCAAAACGTTTGCAACTGCTGCG |

Primer description: Containing exchange paired bases, enzyme cleavage site, and part of the 5' end sequence of the target gene for PCR fishing for the target gene.

2.1 **PCR results**

Size of PCR products：1052

**3. Recombinant plasmid construction**

3.1 **Exchange of products into linearised expression vectors**
3.2 **Primers for PCR identification**

| **ID** | **seq** |
| --- | --- |
| Jun(70082-1)-p1 | GTTTAAACGGGCCCTCTAGACGCCACCATGACTGCAAAGATGGAAAC |
| Jun(70082-1)-p3 | TTATTAGGAAAGGACAGTGGG |

3.3 **PCR identification of recombinant clones**

Positive transformant PCR product size：1158

**4. Sequencing results of positive clones and analysis of results：**

**Comparison results：**

CTGCACCTCGGTTCTGCTAGCGTTTAAACGGGCCCTCTAGACGCCACCATGACTGCAAAGATGGAAACGACCTTCTACGACGATGCCCTCAACGCCTCGTTCCTCCAGTCCGAGAGCGGTGCCTACGGCTACAGTAACCCTAAGATCCTAAAACAGAGCATGACCTTGAACCTGGCCGACCCGGTGGGCAGTCTGAAGCCGCACCTCCGCGCCAAGAACTCGGACCTTCTCACGTCGCCCGACGTCGGGCTGCTCAAGCTGGCGTCGCCGGAGCTGGAGCGCCTGATCATCCAGTCCAGCAATGGGCACATCACCACTACACCGACCCCCACCCAGTTCTTGTGCCCCAAGAACGTGACCGACGAGCAGGAGGGCTTCGCCGAGGGCTTCGTGCGCGCCCTGGCTGAACTGCATAGCCAGAACACGCTTCCCAGTGTCACCTCCGCGGCACAGCCGGTCAGCGGGGCGGGCATGGTGGCTCCCGCGGTGGCCTCAGTAGCAGGCGCTGGCGGCGGTGGTGGCTACAGCGCCAGCCTGCACAGTGAGCCTCCGGTCTACGCCAACCTCAGCAACTTCAACCCGGGTGCGCTGAGCAGCGGCGGTGGGGCGCCCTCCTATGGCGCGGCCGGGCTGGCCTTTCCCTCGCAGCCGCAGCAGCAGCAGCAGCCGCCTCAGCCGCCGCACCACTTGCCCCAACAGATCCCGGTGCAGCACCCGCGGCTGCAAGCCCTGAAGGAAGAGCCGCAGACCGTGCCGGAGATGCCGGGAGAGACGCCGCCCCTGTCCCCTATCGACATGGAGTCTCAGGAGCGGATCAAGGCAGAGAGGAAGCGCATGAGGAACCGCATTGCCGCCTCCAAGTGCCGGAAAAGGAAGCTGGAGCGGATCGCTCGGCTAGAGGAAAAAGTGAAAACCTTGAAAGCGCAAAACTCCGAGCTGGCATCCACGGCCAACATGCTCAGGGAACAGGTGGCACAGCTTAAGCAGAAAGTCATGAACCACGTTAACAGTGGGTGCCAACTCATGCTAACGCAGCAGTTGCAAACGTTTTGATCTAGATAGTTAAACCGCTGATCAGCCTCGACTGTGCCTTCTAGTTGCCAGCCATCTGTTG

**Comparison result description: the sequencing reads through without any errors**
